# The driver role of JAK‐STAT signalling in cancer stemness capabilities leading to new therapeutic strategies for therapy‐ and castration‐resistant prostate cancer

**DOI:** 10.1002/ctm2.978

**Published:** 2022-07-31

**Authors:** U‐Ging Lo, Yu‐An Chen, Junjie Cen, Su Deng, Junghang Luo, Haiyen Zhau, Lin Ho, Chih‐Ho Lai, Ping Mu, Leland W.K. Chung, Jer‐Tsong Hsieh

**Affiliations:** ^1^ Department of Urology University of Texas Southwestern Medical Center Dallas Texas USA; ^2^ Department of Microbiology and Immunology Graduate Institute of Biomedical Sciences College of Medicine Chang Gung University Taoyuan Taiwan; ^3^ Department of Urology First Affiliated Hospital Sun Yat‐sen University Guangdong China; ^4^ Department of Molecular Biology University of Texas Southwestern Medical Center Dallas Texas USA; ^5^ Uro‐Oncology Research Department of Medicine Cedars‐Sinai Medical Center Los Angeles California USA; ^6^ Department of Life Sciences National Chung Hsing University Taichung Taiwan

**Keywords:** IFIT5, interferon signalling, JAK, STAT1, targeted therapy, therapy‐ and castration‐resistant prostate cancer

## Abstract

**Background:**

Lineage plasticity in prostate cancer (PCa) has emerged as an important mechanism leading to the onset of therapy‐ and castration‐resistant PCa (t‐CRPC), which is closely associated with cancer stem cell (CSC) activity. This study is to identify critical driver(s) with mechanism of action and explore new targeting strategy.

**Methods:**

Various PCa cell lines with different genetic manipulations were subjected to in vitro prostasphere assay, cell viability assay and in vivo stemness potential. In addition, bioinformatic analyses such as Ingenuity pathway and Gene Set Enrichment Analysis were carried out to determine clinical relevance. The in vivo anti‐tumour activity of JAK or STAT1 inhibitors was examined in clinically relevant t‐CRPC model.

**Results:**

We demonstrated the role of interferon‐related signalling pathway in promoting PCa stemness, which correlated with significant elevation of interferon related DNA damage resistance signature genes in metastatic PCa. Inhibition of JAK‐STAT1 signalling suppresses the in vitro and in vivo CSC capabilities. Mechanistically, IFIT5, a unique downstream effector of JAK‐STAT1 pathway, can facilitate the acquisition of stemness properties in PCa by accelerating the turnover of specific microRNAs (such as miR‐128 and ‐101) that can target several CSC genes (such as BMI1, NANOG, and SOX2). Consistently, knocking down IFIT5 in t‐CRPC cell can significantly reduce in vitro prostasphere formation as well as decrease in vivo tumour initiating capability.

**Conclusions:**

This study provides a critical role of STAT1‐IFIT5 in the acquisition of PCSC and highlights clinical translation of JAK or STAT1 inhibitors to prevent the outgrowth of t‐CRPC.

## Graphical abstract

Activated JAK‐STAT signalling cascade plays a critical role in the onset of therapy‐ and castration‐resistant disease that is associated with stem cell plasticity. IFIT5, a downstream effector of JAK‐STAT signalling pathway, exhibits a new functional role in modulating stemness transcriptional factors (such as BMI1, NANOG and SOX2) by promoting turnover of specific pre‐miRNAs (such as miR‐101 and miR‐128) for these stemness regulators. Specific JAK‐STAT small molecule inhibitors (ruxolitinib and fludarabine) exhibit potent efficacy in therapy‐ and castration‐resistant prostate cancer treatment.

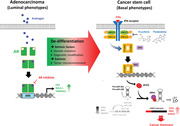

## 1 BACKGROUND

Androgen deprivation therapy (ADT) remains to be the primary therapeutic strategy for hormone naïve metastatic prostate cancer (PCa).^1^ Despite initial profound therapeutic efficacy, the onset of therapeutic‐ and castration‐resistant (t‐CRPC) phenotype becomes inevitable. Data derived from clinical specimens, cell model or transgenic mouse models suggest that distinct genetic alterations^2^, ^3^ are associated with t‐CRPC, which are key contributors to lineage plasticity of PCa because these cells often display neuroendocrine‐like phenotypes.^4^ In general, normal stem cell is an ingredient component in lineage plasticity, however, cancer stem cell (CSC) population not only possess self‐renewal properties and trans‐differentiate into different lineages^5^ but also loose homeostatic regulation. For example, the presence of CSC can be related with cancer recurrence, metastasis and therapeutic resistance.^6^, ^7^, ^8^ In addition to genetic alterations,^9^ some paracrine factors or cytokines have been shown to promote de‐ or trans‐differentiation of PCa cells,^10^ implying the inductive nature of PCa CSC (PCSC). In addition, significant elevation of serum interferon (IFNγ) was found in PCa patients after ADT^11^ and IFN related DNA damage resistance signature (IRDS) genes have been associated with the progression of many malignancies.^12^, ^13^

Moreover, both signal transducer and activator of transcription 1 (STAT1) and STAT2 are essential regulators in the signalling cascaded stimulated by either type I (IFN‐α, IFN‐β) or type II (IFN‐γ) IFNs. Upon IFN stimulation, Janus kinases (JAK)‐mediated phosphorylation of STAT leads to its activation and nuclear translocation. Activated STAT1 or STAT2 binds to the IFN‐stimulated response element (ISRE) in the promoter region and induce transcriptional activation of IFN‐stimulated genes (ISGs). Particularly, IFIT5, a bona fide IFN‐inducible gene which is initially recognized as a viral RNA‐binding protein in the immune mechanism against virus infection, has been revealed to perform pro‐tumorigenic functions. In our study, we observed that IFIT5 is able to facilitate PCa epithelial‐to‐mesenchymal transition (EMT)^14^ that is associated with CSC potential.^15^ Nevertheless, the role of IFN in PCSC development is largely unknown.

In this study, we demonstrate that activation of JAK‐STAT1 pathway in PCa cells treated with IFN or anti‐androgen are the potent upstream driver pathway in eliciting PCSC activities toward neuroendocrine (NE) cell lineage via induction of IFIT5‐mediated microRNA turnover machinery. Specifically, we have unveiled the underlying mechanism of action of IFIT5 in promoting PCSC activities and identified clinically available STAT1 or JAK inhibitor as potential targeted therapeutics for preventing t‐CRPC progression.

## 2 METHODS

### 2.1 Cell lines and gene transfection

Cells obtained from ATCC (Manassas, VA): LNCaP and DU145 cells are maintained in RPMI‐1640 medium supplemented with 10% foetal bovine serum (FBS), and CWR‐22Rv1 cells were maintained in DMEM medium supplemented with 10% FBS. C4‐2 and ARCaP wild type (WT)^16^ and single cell sublines (IIIF9, IIF11, Fast, IIB5, IIID5 and IIG5) were maintained in RPMI‐1640 medium supplemented with 10% FBS. C4‐2B parental (with the same medium as C4‐2) and C4‐2B MDVR cell lines are given by Dr. Allen Gao (UC Davis, CA)^17^; C4‐2B MDVR line was cultured in Phenol Red free RPMI‐1640 medium supplemented with 10% charcoal stripped‐FBS (CS‐FBS) plus 20 μM ENZ. LNCaP‐sgNT (CRISPR‐control) and LNCaP‐sgTP53/RB1 (CRISPR‐TP53 and RB1‐knockout) cell lines were established by Dr. Mu.^3^ Stable IFIT5‐shRNA knockdown (KD) (shIFIT5) and control (shCon) prostate cell lines were generated from DU145 and LNCaP sgTP53/RB1 cell lines using pLKO‐shIFIT5 plasmid generated by Academia Sinica, Taipei, Taiwan. Stable IFIT5‐overexpressing (IFIT5) and control (Vec) cell lines were generated from 22Rv1 and LNCaP cell lines using pcDNA3.1‐3XFlag‐IFIT5 plasmid provided by Dr. Collins (UC Berkeley, CA). All these cell lines were authenticated with the short tandem repeat (STR) profiling by Genomic Core in UT Southwestern Medical Center (UTSW) periodically and mycoplasma testing was performed by MycoAlert® kit (Lonza Inc. Walkersville, MD) to ensure mycoplasma‐free condition.

Cells (2.5 × 10^5^) were seeded in 60‐mm dish at 60%–70% confluence before transfection. According to manufacturer's protocol, transfection of plasmids was using either Xfect Reagent (Clontech) or EZ Plex transfection reagent (EZPLEX). Transient transfection was carried out 48 h post‐transfection to harvest cell for further analyses. In addition, the stable clones were established after 3 weeks of culture at the antibiotic selective medium.

### 2.2 Construction of SSMut and DSMut precursor miRNA‐expression vector

Native miR‐128 or miR‐101 expressing plasmid (Genecopoeia) was used as a template to generate mutant pre‐miR‐128 with 5′‐six nucleotides single stranded overhang (pre‐SS6Mut‐miR‐128) or double‐stranded blunt end (pre‐DSMut‐miR‐128), as well as mutant pre‐miR‐101 with 5′‐nine nucleotides single stranded overhang (pre‐SS9Mut‐miR‐101) or double‐stranded blunt end (pre‐DSMut‐miR‐101) constructs using site‐directed mutagenesis.

### 2.3 RNA isolation, RNA‐sequencing (RNA‐seq) and quantitative real‐time RT‐PCR (qRT‐PCR)

Total RNA was isolated and purified using Maxwell® 16 miRNA Tissue kit or Maxwell® 16 LEV Simply RNA Tissue Kit (Promega). Small RNA from total RNA (2 μg) was subjected to miScript II RT kit (QIAGEN) then 2.5 μl cDNA was applied to a 25‐μL reaction volume using miScript SYBR Green PCR kit (QIAGEN) in iCycler thermal cycler (Bio‐Rad). The relative expression levels of matured miRNAs from each sample were determined by normalizing to SNORD95 small RNA. Large RNA from total RNA (2 μg) was subjected to iScriptTM Adv cDNA kit (BioRad) then 2.5 μl cDNA was applied to a 25‐μL reaction volume using iTaq Universal SYBR® Green Supermix (BioRad) in iCycler thermal cycler (Bio‐Rad). All primer sequences are listed in Table S5. The relative expression levels of IFIT5, STAT1, BMI1, NANOG, SOX2, IFI44, IFI44L, IFI6, IFIT1, IFIT3, IFITM1, MX1, MX2, OAS1 and OAS3 mRNA from each sample were determined by normalising to 18S mRNA. All quantitative data of miRNA or mRNA expression level were analysed using ΔCt (Ct value normalised to internal snord95 miRNA or 18S RNA) and ΔΔCt (difference between the ΔCt of control and experimental groups) values to obtain the fold change after normalising with control group.

RNA‐Seq was performed by Novogene. Briefly, RNA libraries were prepared from 500 ng of total RNA using NEBNext^®^ Ultra™ kit then subjected to NovaSeqTM 6000 Sequencing System (illuminea). FPKMs for genes and transcripts were generated by StringTie (v1.3.5), and RSeQC (v3.0.0) was used for generating RNA quality control metrics. Differential gene expression analysis was performed using the R package DEseq2 (v1.6.3). Cut‐off values of absolute fold change greater than two and FDR < 0.1 were used to select for differentially expressed genes between sample group comparisons.

### 2.4 Determination of IFIT5 gene promoter activities

The IFIT5 gene promoter‐driven mCherry reporter construct^14^ was transfected into LNCaP cells to generate the stable clones. Established stable clones were plated on 2D monolayer cultural condition or 3D sphere ultra‐low attachment plate. The IFIT5 gene promoter activities were determined based on the expression of mCherry protein under fluorescent microscopy.

### 2.5 Western blot analysis

Cells were lysed in lysis buffer [50mMTris‐HCl (pH 7.5), 150 mM NaCl, 0.1% Triton X‐100, 1 mM sodium orthovanadate, 1 mM sodium fluoride, 1 mM sodium pyrophosphate, 10 mg/mL, aprotinin, 10 mg/mL leupeptin, 2 mM phenylmethylsulphonyl fluoride, and 1 mM EDTA] for 30 min on ice. Cell lysates were spin down at 20 000 xg for 20 min at 4°C. Protein extracts were subjected to SDS‐PAGE using Bolt 4%–12% Bis‐Tris Plus gel (Invitrogen) and transferred to nitrocellulose membrane using Trans‐Blot Turbo Transfer system (BIORAD). Membranes were incubated with primary antibodies against IFIT5, NANOG (ProteinTech), BMI1, SOX2, STAT1, JAK1 (Cell Signalling Technology), as well as GAPDH (Santa Cruz Biotechnology) at 4°C for 16–18 h, and horseradish peroxidase‐conjugated secondary antibodies at room temperature for 2 h. Results were visualised with ECL chemiluminescent detection system (Pierce ThermoScientific). The relative protein expression level in each sample was normalized to GAPDH.

### 2.6 In vitro prostasphere formation and clonogenic assay

Cells were counted and seeded into 96‐well ultra‐low attachment plate (Corning) at density of 250 cells/per well in DMEM (Gibco) supplemented with B‐27 supplement (Gibco), recombinant human basic Fibroblast Growth Factor (10 ng/ml bFGF, Gibco) and human Epidermal Growth Factor (20 ng/ml hEGF, Gibco). Inhibitors (fludarabine or ruxolitinib) or interferons were added to the culture medium prior to seeding the cells. Formation of sphere was observed and quantified under microscope at week 1 or 2 after seeding.

For clonogenic assays, 100 μL of cells (300 cells/well) was mixed with 100 μL of cold Matrigel and then plated around the rim of a 24‐well dish. After solidification at 37°C for 15 min, 200 mL warm PrEBM was added in the centre of the dish. Colonies were enumerated in 1 to 2 weeks.

### 2.7 In vivo tumour incidence

All animal works were approved by the Institutional Animal Care and Use Committee from UTSW. Serial dilutions of stable shCon or shIFIT5 KD DU145 cells mixed 1:1 with Matrigel (BD Biosciences) were injected subcutaneously into male SCID (6 weeks‐old) mice. The incidence of DU145 tumour was determined 8 weeks after injection followed by measurement of tumour weights and tumour volumes. The tumour volume was determined by calliper and calculated (length × width × width)/2.

### 2.8 In vivo experimental therapy study

Initially, ARCaP‐F11 (1.5 × 10^6^) or LNCaP sgTP53/RB1 (5 × 10^6^) or 22Rv1 (1 × 10^6^) cells mixed 1:1 with Matrigel (BD Biosciences) were injected subcutaneously into both flanks of SCID (6 weeks‐old) mice. When tumour volume reached 100 mm^3^, mice were then randomly distributed into three groups: Vehicle (10% DMSO, i.p.), fludarabine (20 mg/kg, i.p.) or ruxolitinib (20 mg/kg, i.p.) was administered 5 days per week for 2 weeks. Tumour growth rate was measured at Day 0, 4, 7, 11, 14, 18, 21 and 25 after treatment. The average tumour volumes of each group were calculated according to the equation of volume = (length × width × width)/2.

### 2.9 In vivo bone metastasis model

ARCaP‐Fast (1.5 × 10^6^) cells transfected with luciferase plasmid were injected into the right ventricle of SCID mouse via ultrasound guided intra‐cardiac injection. The metastatic tumour nodules were monitored by bioluminescence imaging (BLI) after 6–8 weeks post‐injection. Tumours were excised from spinal vertebrae, then established the ARCaP‐Fast metastatic subline.

### 2.10 Statistics analysis

Statistics analyses were performed by using GraphPad Prism software. Statistical significance was evaluated using Student *t*‐test. *p < .05, **p < .001 or ***p < .0001 was considered a significant difference between compared groups and marked with asterisks.

## 3 RESULTS

### 3.1 Clinical prevalence of STAT1 in metastatic PCa is associated with PCSC phenotypes

**FIGURE 1:**
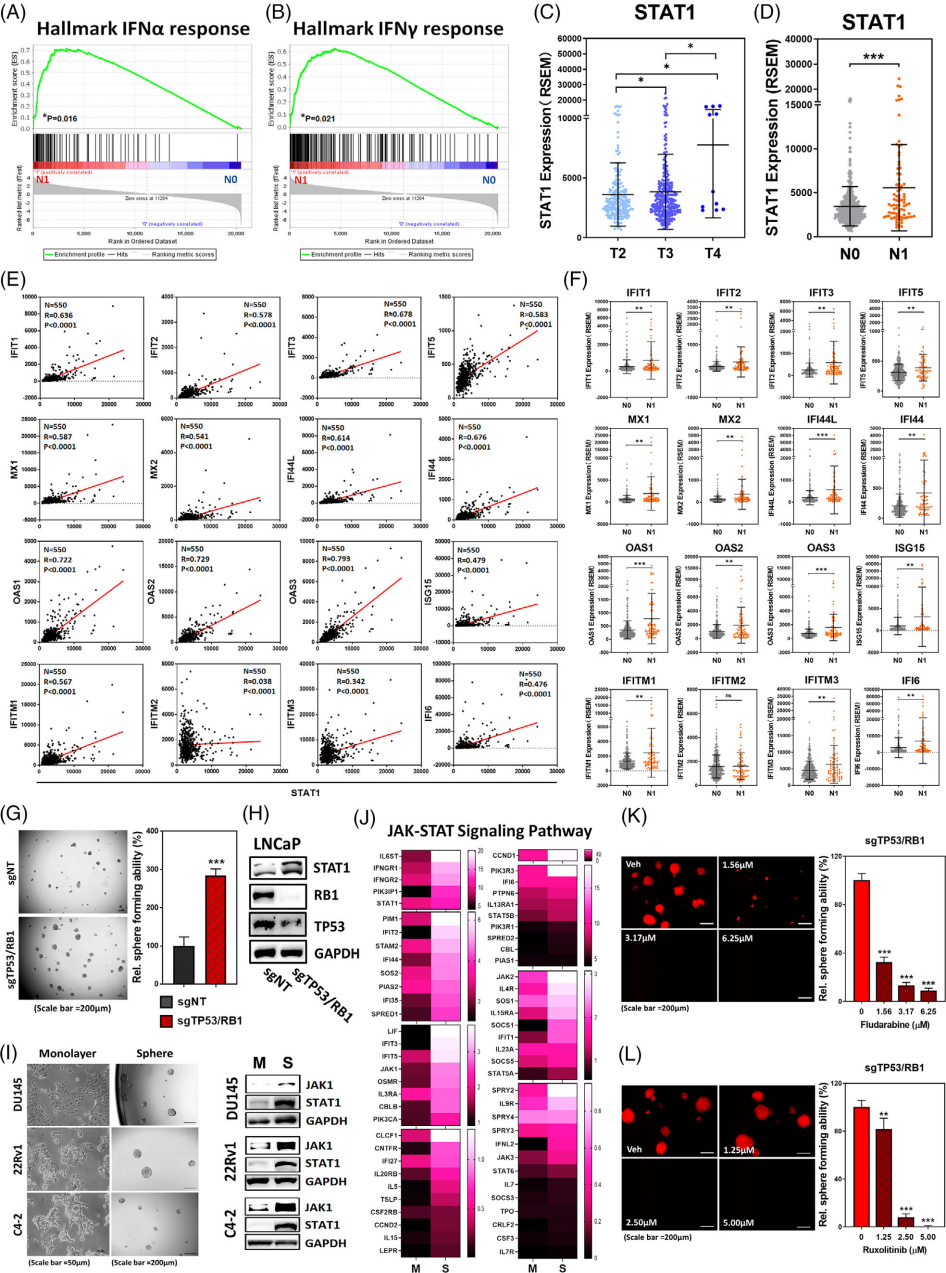
Emergence of STAT1 in advanced PCa contributes to the acquisition of self‐renewal capacity in PCSCs. (A,B) GSEA identifying upregulation of genes associated with IFNα and IFNγ response in the tumour specimens of PCa patients with lymph node metastasis (N1, Red, *n* = 80), compared to cohort without metastasis (N0, Blue, *n* = 345). (C) TCGA PCa dataset demonstrating STAT1 expression level among stage T2 (*n* = 187), T3 (*n* = 293) and T4 (*n* = 11) PCa patients. (D) TCGA PCa dataset demonstrating elevation of STAT1 in PCa patients with lymph node metastasis (N1, *n* = 80), compared to cohort without metastasis (N0, *n* = 345). (E) Clinical correlation of STAT1 with interferon response‐associated genes (*N* = 550) derived from TCGA PCa dataset was calculated using Pearson Correlation. (F) Expression level of interferon response‐associated genes in PCa patients with lymph node metastasis (N1, *n* = 80), compared to cohort without metastasis (N0, *n* = 345). (G) The sphere forming ability of sgTP53/RB1‐LNCaP cell, compared to vector control (sgNT). (H) Protein level of STAT1, RB1 and TP53 in sgTP53/RB1‐LNCaP cell, compared to vector control (sgNT). (I) Left: morphology of DU145, 22Rv1 and C4‐2 cells under adherent monolayer or ultra‐low attachment sphere culture condition. Right: JAK1 and STAT1 protein expression in the sphere (S) derived from each PCa line, compared to corresponding monolayer (M) culture. (J) Heat map illustrating the expression profile of JAK‐STAT signalling pathway molecules in the sphere (S) derived from LNCaP cells, compared to monolayer (M) adherent culture. (K,L) Dose‐dependent impact of fludarabine and ruxolitinib on the sphere forming ability of sgTP53/RB1‐LNCaP cells. Cells were treated with either fludarabine or ruxolitinib at corresponding concentration right after seeding into the ultra‐low attachment plate. Quantitation of sphere forming ability is done at 2 weeks afterward. (ns = no significant differences, *p < .05, **p < .001, ***p < .0001)

Gene set enrichment analysis (GSEA) of TCGA PCa dataset (Figure 1A,B) indicated that either type I or II IFN‐induced genes (Figure S1A) are up‐regulated in PCa patients with lymph node metastasis (N1), compared to none‐metastasis cohort (N0). IRDS genes including a subset of STAT1‐driven genes, have been associated with the progression of many malignancies,^12^, ^13^ and are significantly up‐regulated in N1 PCa patients (Figure S1B). STAT1 is a central transcriptional factor activated by IFN stimulation. Based on the TCGA PCa Dataset, significant elevation of STAT1 level is detected in PCa patients with either higher stage (Figure 1C), lymph node metastasis (Figure 1D), or higher grade (Figure S1C) as well as in other malignancies (Figure S1D). However, when compared to normal tissue, STAT1 has no significant elevation in PCa tumour (Figure S1D). We further explored the relationship between IFN‐regulated downstream effectors and the status of STAT1 gene expression in PCa patients and found significant correlation from most of the effectors (Figure 1E), which are also elevated in N1 PCa patients (Figure 1F). These data support the role of IFN‐STAT1 signalling pathway in PCa progression.

Loss of TP53 and RB1 increases SOX2 expression that is associated with the lineage plasticity of PCa cells.^3^, ^9^ To demonstrate PCSC capabilities using prostasphere forming assay,^18^ we observed that prostasphere formation of sgTP53/RB1 LNCaP cells is significantly increase compared to sgNT control (Figure 1G). Furthermore, we checked other PCSC‐associated markers and observed significant upregulation of CD44, CD133, CD49f and TROP2 in sgTP53/RB1 LNCaP cells (Figure S1E), and this evidence further supported its PCSC phenotypes. Moreover, elevated STAT1 levels are detected in the sgTP53/RB1 LNCaP cells (Figure 1H), while both JAK1 and STAT1 are increased in the prostaspheres (S) derived from several PCa lines compared with monolayer culture (M) (Figure 1I and Figure S1F). Indeed, in contrast to STAT1 (Figure 1C and Figure S1C), TP53 level is significantly reduced in PCa patients with higher grade (Figure S1G) or lymph node metastasis (Figure S1H), and inversely correlated with STAT1 level based on TCGA PCa dataset (Figure S1I). Based on RNA‐seq analysis, genes involved in JAK‐STAT1 signalling cascade are highly upregulated in the prostasphere of LNCaP cells, compared to monolayer adherent culture (Figure 1J). Hence, we further examined the role of STAT1 and its upstream regulator‐JAK in prostasphere formation using small molecule inhibitors (SMIs) fludarabine^19^ and ruxolitinib^20^ specifically targeting STAT1 and JAK1 or JAK2, respectively. Both SMIs significantly suppressed prostasphere formation of sgTP53/RB1 LNCaP (Figure 1K,L), DU145 (Figure S1J,K), and 22Rv1 (Figure S1L,M) cells, indicating JAK‐STAT1 signalling is critical for the acquisition of stemness properties in PCa.

### 3.2 IFN‐elicited signalling accelerates the development of NE phenotypes during PCa progression

ARCaP line is initially characterized as AR‐repressive PCa cells derived from a patient with ascites metastasis after failure of ADT; apparently, this cell line contains heterogeneous cell population based on morphology and expression profile of biomarker.^16^, ^21^ Therefore, we determine to analyse the gene expression profile of single cell clones of ARCaP by RNA‐seq.

**FIGURE 2:**
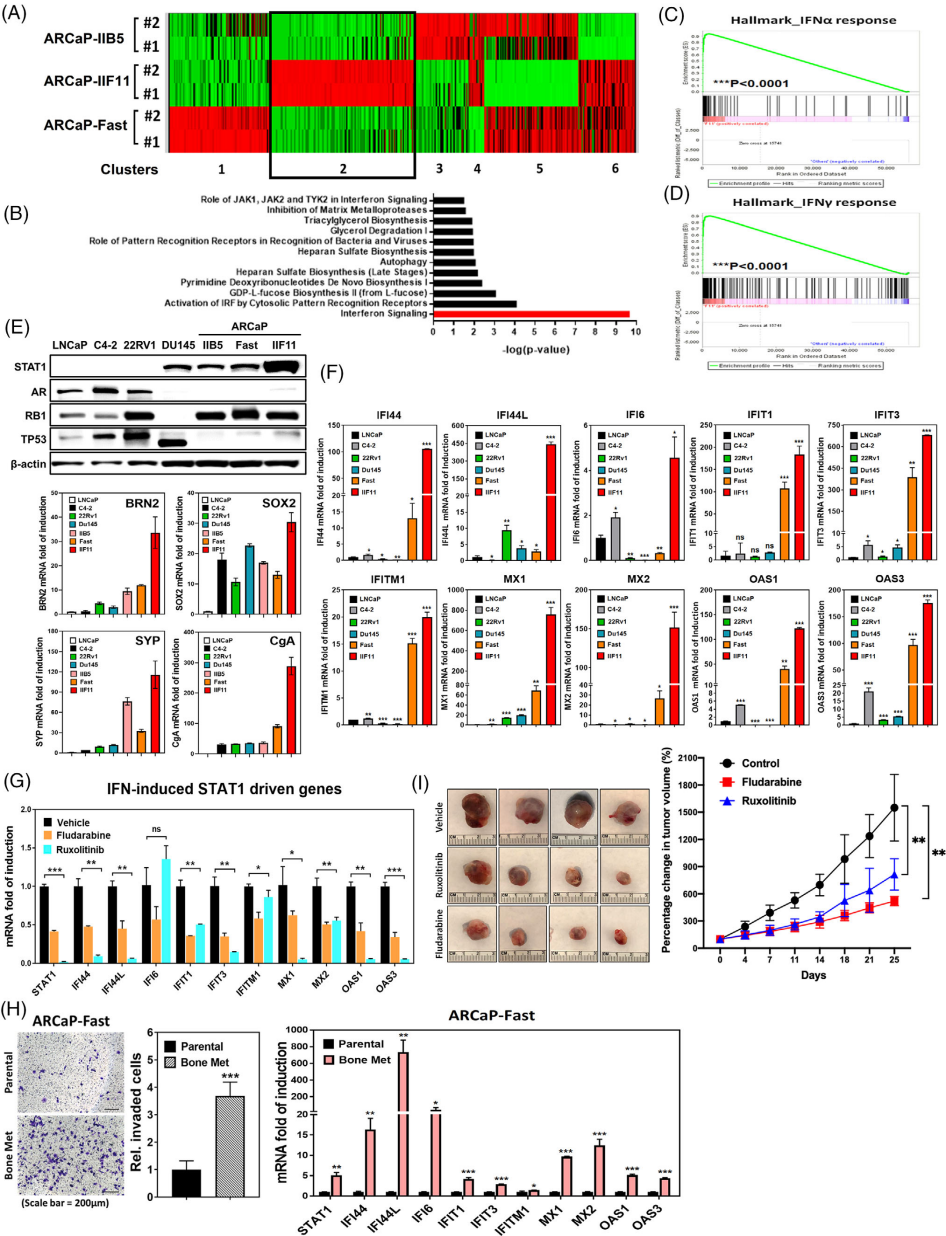
STAT1‐IFIT5 signalling activation emerges during the advanced progression of PCa toward CRPC and NEPC. (A) Heat map illustrating the gene expression profile among three ARCaP sublines‐IIB5, IIF11 and Fast. (B) The ingenuity pathway analysis of significant enrichment of IFN signalling pathway genes in ARCaP‐IIF11, compared to ARCaP‐Fast and ‐IIB5 sublines. (C,D) GSEA identifying enrichment of genes associated with IFNα and IFNγ response in IIF11 subline, compared to Fast and IIB5 sublines. (E) Upper: expression of STAT1, AR, RB1 and TP53 protein levels among screened PCa lines including ARCaP sublines. Lower: screening of BRN2, SOX2, SYP and CgA mRNA levels among PCa lines including ARCaP sublines. (F) Expression level of 10 IFN‐inducible STAT1‐driven genes among PCa lines including ARCaP sublines. (G) Expression of ten IFN‐inducible STAT1‐driven genes in ARCaP‐IIF11 subline treated with fludarabine (1 μM) or ruxolitinib (2.5 μM) for 48 h. (H) Left: expression of ten IFN‐inducible STAT1‐driven genes in ARCaP‐Fast bone metastatic subline, compared to parental line. Right: in vitro invasion assay examining the invasiveness of ARCaP‐Fast bone metastatic line. (I) The impact of fludarabine (20 mg/kg) or ruxolitinib (20 mg/kg) on the sub‐cutaneous ARCaP‐IIF11 tumour growth, compared to vehicle control. (ns = no significant differences, *p < .05, **p < .001, ***p < .0001)

In comparison to the adenocarcinoma line, the t‐CRPC ARCaP sublines showed differential expression profile of androgen‐responsive genes that are mostly downregulated when compared to LNCaP line (Figure S2A). On the other hand, genes involved in JAK‐STAT signalling cascade are highly elevated in ARCaP sublines (Figure S2B). Similar outcome is also observed when we examine the expression profile of genes responsive to IFNγ stimulation (Figure S2C). Based on this observation, six gene clusters were categorized by their distinct function in three ARCaP sublines (IIF11, Fast and IIB5) (Figure 2A). In particular, the ingenuity pathway analyses demonstrate that cluster two genes associated with IIF11 subline are highly involved in the IFN signalling pathway (Figure 2B). Indeed, GSEA confirms that IIF11 subline has elevated expression of genes involved in IFNα (Figure 2C and Table S1) and IFNγ response (Figure 2D and Table S1). Moreover, based on gene expression profile among PCa cell lines, neuroendocrine transcriptional factor (BRN2 and SOX2) and biomarkers (SYP and CgA) are related with STAT1 protein level that is highly elevated in ARCaP‐IIF11 subline (Figure 2E). Expression level of STAT1 among examined PCa lines are also inversely correlated with AR level (Figure 2E). Similar relationship was observed in sgTP53/RB1 LNCaP exhibiting neuroendocrine phenotypes (Figure S2D)^3^ or castration resistant C4‐2B MDVR^17^ (Figure S2E); targeting STAT1 by fludarabine is able to suppress these genes in C4‐2B MDVR line (Figure S2F). On the other hand, IFNβ can enhance upregulation of these genes in DU145 cells (Figure S2G). These data support the inductive role of IFN in neuroendocrine differentiation of PCa cells.

Furthermore, RNA‐seq result indicated that 18 out of 45 IRDS genes are significantly higher in IIF11 than Fast or IIB5 subline (Table S2). Among these 18 IRDS genes, a subset of 10 STAT1‐driven genes (IFI44, IFI44L, IFI6, IFIT1, IFIT3, IFITM1, MX1, MX2, OAS1 and OAS3) are positively correlated with STAT1 in clinical TCGA PCa dataset (Figure 1E) and elevated in PCa patients with lymph node metastasis (Figure 1F). Therefore, we determine to examine these ten IFN‐induced STAT1‐driven genes among PCa lines. Indeed, IIF11 subline has the highest expression level of all ten genes among examined PCa lines (Figure 2F). On the other hand, treatment with either fludarabine or ruxolitinib results in significant downregulation of all ten genes in IIF11 subline (Figure 2G), suggesting they are downstream to JAK‐STAT1 signalling axis. Meanwhile, the levels of STAT1, IFI44, IFI6, IFIT1, IFIT3, MX1, MX2, OAS1 and OAS3 expression were significantly higher in DU145 and 22Rv1 prostaspheres than monolayer culture condition (Figure S2H). Upregulation of STAT1 and STAT1‐driven genes was also observed in bone metastatic subline with invasiveness (Figure 2H) derived from ARCaP‐Fast tumour (Figure S2I). Thus, i.p. administration of fludarabine (20 mg/kg) or ruxolitinib (20 mg/kg) into mice bearing subcutaneous ARCaP‐IIF11 tumours, we observed significant growth inhibition compared to the vehicle control (Figure 2I). Apparently, STAT1 represents a potent therapeutic target for t‐CRPC. Overall, these data demonstrate the activation of JAK‐STAT1 signalling contributes to the acquisition of lineage plasticity of CRPC lines.

### 3.3 STAT1 signalling‐induced IFIT5 upregulation facilitates the acquisition of stemness properties in PCa

**FIGURE 3:**
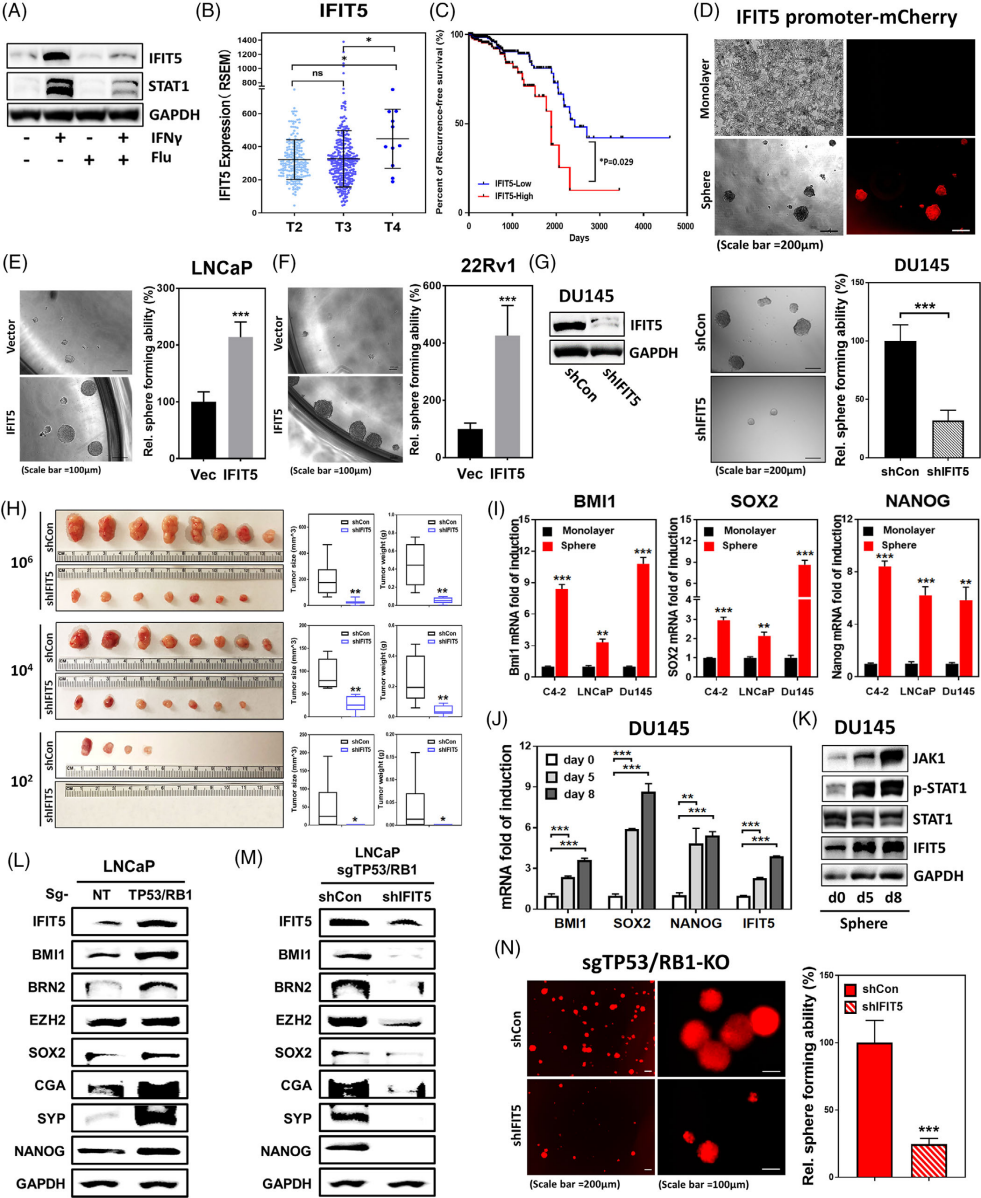
STAT1‐mediated induction of IFIT5 facilitates the emergence of PCSC. (A) Impact of fludarabine‐mediated STAT1 inhibition on IFNγ‐induced IFIT5 protein upregulation in DU145 cell. (B) TCGA PCa dataset demonstrating IFIT5 expression level among Stage T2 (*n* = 187), T3 (*n* = 293) and T4 (*n* = 11) PCa patients. (C) Kaplan–Meier survival curve of PCa patients grouped into IFIT5‐high and IFIT5‐low cohorts analysed by long‐rank test (*p* = .029). (D) Expression of IFIT5 promoter‐driven mCherry fluorescent protein in tumour sphere derived from LNCaP cells, compared to monolayer adherent culture. (E,F) The impact of IFIT5‐OE on sphere forming ability of LNCaP and 22Rv1 cells, compared to vector control (Vec). (G) IFIT5 KD in DU145 line and its impact on the prostasphere forming ability of DU145 cells, compared to control shRNA (shCon). (H) Quantified size and weight of subcutaneous IFIT5‐KD DU145 tumours, compared to shCon cohort. (I) Expression level of BMI1, SOX2 and Nanog mRNA in spheres derived from DU145, LNCaP and 22Rv1 cell lines, compared to each corresponding monolayer adherent culture. (J) Induction of BMI1, SOX2, NANOG and IFIT5 gene upregulation during DU145 prostasphere formation. (K) Induction of JAK1, phospho‐STAT1, STAT1 and IFIT5 protein upregulation during DU145 prostasphere formation at days 5 and 8 post‐seeding. (L) Protein expression of IFIT5, BMI1, BRN2, EZH2, SOX2, CgA, SYP and Nanog in sgTP53/RB1‐LNCaP cell, compared to control vector (sgNT). (M) Protein level of IFIT5, BMI1, BRN2, EZH2, SOX2, CgA, SYP and Nanog in IFIT5‐KD (shIFIT5) sgTP53/RB1‐LNCaP cells, compared to control vector (shCon). (N) Reduction of prostasphere formation in IFIT5‐KD sgTP53/RB1‐LNCaP cells, compared to control vector (shCon) (ns = no significant differences, *p < .05, **p < .001, ***p < .0001)

In response to IFNγ treatment, we observed a significant induction of IFIT5 (Figure S3A), which is abolished by STAT1 KD (Figure S3B) or fludarabine treatment (Figure 3A). The dose‐dependent inhibitory effect of fludarabine on IFIT5 induction is also observed in PC3 cells treated with IFNβ (Figure S3C). Clinically, elevated IFIT5 is associated with PCa progression (Figures 1F and 3B) and predicts poor recurrence‐free survival in PCa patients (Figure 3C). A positive correlation between STAT1 and IFIT5 seen in PCa (Figure 1E) is also observed in breast and brain malignancies (Figure S3D). Moreover, elevated IFIT5 gene promoter activities (Figure 3D), mRNA (Figure S3E) and protein (Figure S3F) is associated with prostaspheres formation. Based on IFIT5 expression profile in several PCa lines (Figure S3G), IFIT5 expression vector was transfected into either LNCaP or 22Rv1 cells (Figure S3H) and the result indicated that IFIT5 significantly facilitates the number and size of prostasphere formation of both cells (Figure 3E,F). In contrast, loss of IFIT5 in DU145 cells leads to significantly lower number of prostasphere formation and impacts on its clonogenicity when compared to vector control (Figure 3G and Figure S3I). Consistent with in vitro observation, the incidence of DU145 tumorigenesis is significantly reduced by IFIT5 loss (Figure 3H and Figure S3J), which is similar with their tumour size and weight (Figure 3H; Tables S3 and S4). Overall, IFIT5, a bona fide IFN‐STAT1‐inducible gene, is critical for maintaining PCSC capabilities.

### 3.4 IFIT5 modulates BMI1, SOX2 and NANOG gene expression underlying PCSC capabilities

Among numerous CSC‐associated genes, Polycomb complex protein Bmi‐1 (BMI1),^22^ SOX2^23^ and NANOG^24^ have been associated with human PCSC activities. We found, like IFIT5, these three genes are significantly elevated in prostaspheres derived from several PCa lines (Figure 3I). The presence of IFIT5 in 22Rv1 or LNCaP line can induce upregulation of BMI1, SOX2 and NANOG (Figure S3K,L). In contrast, IFIT5 loss not only decreases prostasphere formation significantly (Figure 3G) but also downregulates BMI1, SOX2, and NANOG in DU145 cells (Figure S3M). Meanwhile, there is an apparent time‐dependent mRNA upregulation among BMI1, SOX2, NANOG and IFIT5 during prostasphere formation (Figure 3J and Figure S3N,O), which is paralleled with JAK‐STAT1 signalling activation (Figure 3K). Also, the significant elevation of TROP2, CD49f, CD44 and CD133 mRNA level representing PCSC biomarkers, is observed in IFIT5‐overexpressing LNCaP cells (Figure S3P). In addition to SOX2 elevation in sgTP53/RB1 LNCaP cells, both BMI1 and NANOG mRNA levels were elevated (Figure S3Q), as expected, elevated SOX2, BMI and NANOG protein levels were observed in sgTP53/RB1 LNCaP cells, along with increased expression of IFIT5, EZH2, CgA and SYP (Figure 3L). On the other hand, BMI1, SOX2 and NANOG along with BRN2, EZH2, CgA and SYP were significantly downregulated at protein level in IFIT5 KD sgTP53/RB1 LNCaP cells (Figure 3M), which is closely associated with its significantly decreased prostasphere formation (Figure 3N).

### 3.5 The mechanism of IFIT5‐elicited PCSC activities is mediated by specific precursor microRNA (pre‐miRNA) turnover

**FIGURE 4:**
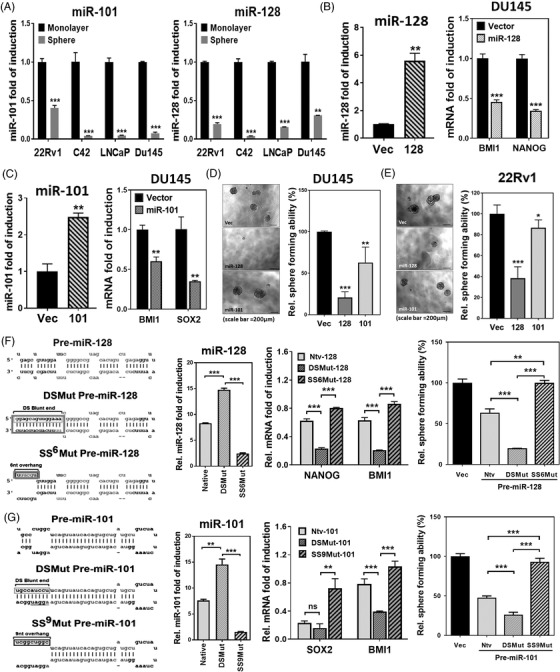
IFIT5 promotes the expression of PCSC‐associated regulators via targeting miRNA biogenesis at precursor level (A) Expression level of miR‐101 and miR‐128 in spheres derived from 22Rv1, C4‐2, LNCaP and DU145 cell lines, compared to each corresponding monolayer adherent culture. (B) The impact of miR‐128 OE on Nanog and Bmi1 level in DU145 cells, compared to vector control. (C) The impact of miR‐101 OE on SOX2 and BMI1 level in DU145 cells, compared to vector control. (D, E) The impact of miR‐128 and miR‐101 on the tumour sphere formation of DU145 and 22Rv1 cells. (F) Left: mutation of nucleotides (box) for generating blunt 5′‐end double stranded pre‐miR‐128 (DSMut pre‐miR‐128) or 5′‐end six nucleotides single stranded pre‐miR‐128 (SS6Mut pre‐miR‐128). Both mature miR‐128 and miR‐128* sequences were shown in lighter grey. Middle: the effect of Native, DSMut, or SS6Mut pre‐miR‐128 on the expression level of mature miR‐128 in DU145 cells. The impact of Native, DSMut, or SS6Mut pre‐miR‐128 on the expression level of NANOG and BMI1 mRNA in DU145 cells. Right: the impact of Native, DSMut, or SS6Mut pre‐miR‐128 on the sphere formation of DU145 cells. (G) Left: mutation of nucleotides (box) for generating blunt 5′‐end double stranded pre‐miR‐101 (DSMut pre‐miR‐101) or 5′‐end nine nucleotides single stranded pre‐miR‐101 (SSMut pre‐miR‐101). Both mature miR‐101 and miR‐101* sequences were shown in lighter grey. Middle: The effect of Native, DSMut, or SS9Mut pre‐miR‐101 on the expression level of mature miR‐101 in DU145 cells. The impact of Native, DSMut, or SS9Mut pre‐miR‐101 on the expression level of SOX2 and BMI1 mRNA in DU145 cells. Right: The impact of Native, DSMut, or SS9Mut pre‐miR‐101 on the sphere formation of DU145 cells. (ns = no significant differences, *p < .05, **p < .001, ***p < .0001)

IFIT5 is known to regulate viral RNA turnover.^25^, ^26^ Our recent study^14^ unveiled a novel functional role of IFIT5 in degrading a group of pre‐miRNAs with specific end structure. Among the many miRNAs identified to be IFIT5 target, we noticed that miR‐101 and miR‐128 share the similar mRNA targets that are involved in stem cell development. Thus, we examined both miR‐101 and miR‐128 levels in IFIT5‐elicited prostaspheres and found significant downregulation of both mature miRs in prostaspheres derived from different PCa lines (Figure 4A), which appear to be dose‐dependent suppression by IFIT5 overexpression (OE) (Figure S4A). We further investigated whether miR‐128 or miR‐101 can target BMI1, SOX2 or NANOG mRNA. The 3′UTR of BMI1 and NANOG mRNA was predicted to match with the seed sequence of miR‐128 (Figure S4B) and the 3′UTR of SOX2 and BMI1 mRNA was predicted to match with the seed sequence of miR‐101 (Figure S4C). Indeed, miR‐128 OE can decrease BMI1 and NANOG mRNA level (Figure 4B and Figure S4D) and miR‐101 OE is able to decrease BMI1 and SOX2 expression (Figure 4C and Figure S4D). Functionally, the presence of miR‐128 or miR‐101 can inhibit prostasphere formation of DU145 or 22RV1 cell (Figure 4D,E).

To examine if the mechanism of IFIT5‐elicited PCSC activities is mediated by the degradation of both miRNAs, we transfected two mutant forms of pre‐miR‐128 (Figure 4F) and pre‐miR‐101 (Figure 4G) into IFIT5‐expressing DU145 cell; the DSmut can resist IFIT5‐medicated degradation and the SSmut is sensitive to IFIT5‐medicated degradation. Therefore, in the presence of DSmut of each pre‐miR, the significant reduction of corresponding target gene was observed but SSmut did not change or slightly increase the corresponding target gene and its protein expression (Middle panel in Figure 4F,G and Figure S4E,F). As expected, the IFIT5‐resistant DSmut can significantly inhibit prostasphere formation but IFIT5‐sensitive SSmut has an opposite activity (Right panel in Figure 4F,G). Similar observation was found in prostasphere formation of 22Rv1 (Figure S4G) or LNCaP cells (Figure S4H). Overall, these data support that the turnover of precursor miR‐101 or ‐128 modulated by IFIT5 plays a central role in PSCS activities.

### 3.6 IFN potentiates the acquisition of PCSC properties via STAT1‐IFIT5 axis

**FIGURE 5:**
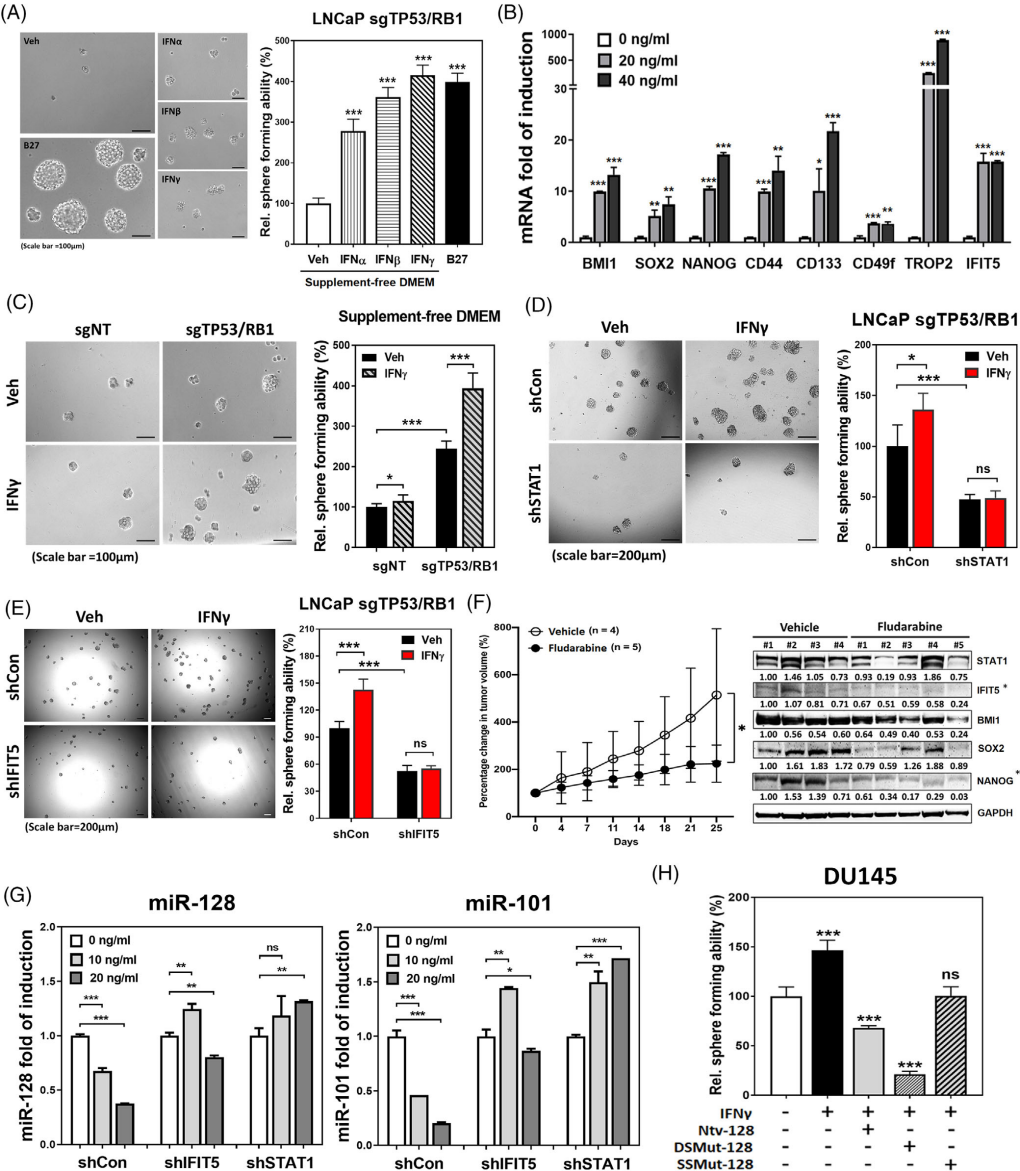
Interferon‐STAT1‐IFIT5 signalling potentiates the acquisition of cancer stemness properties in PCa. (A) The impact of type I and Type II IFN treatment on the sphere formation of sgTP53/RB1 LNCaP cells cultured within supplement‐free DMEM condition. (B) The dose‐dependent impact of IFNγ treatment on the induction of IFIT5 and cancer stemness‐associated genes in 22Rv1 cells. (C) The impact of IFNγ on the self‐renewal capacity of sgTP53/RB1‐LNCaP cells under supplement‐free DMEM culture condition, compared to sgNT control. Cells were treated with 20 ng/ml of IFNγ right after seeding into the ultra‐low attachment plate. Sphere numbers were quantified at 2 weeks after seeding. (D,E) The impact of IFIT5‐ or STAT1‐KD on the IFNγ‐facilitated sgTP53/RB1‐LNCaP sphere formation, compared to control vector (shCon). LNCaP cells were treated with 20 ng/ml of IFNγ right after seeding into the ultra‐low attachment plate. Sphere numbers were quantified at 2 weeks after seeding. (F) Left: the impact of fludarabine (10 mg/kg) on LNCaP sgTP53/RB1 tumour growth. Right: expression of STAT1, IFIT5, BMI1, NANOG and SOX2 protein levels in the shTP53/RB1‐LNCaP tumours treated with fludarabine (10 mg/kg), compared to vehicle control. Statistically, both IFIT5 and Nanog proteins are significantly reduced in the fludarabine treated group, compared to vehicle control. (STAT1 *p* = .34, IFIT5 *p* = .006, BMI1 *p* = .076, SOX2 *p* = .081, NANOG *p* = .005). Fludarabine was administrated through intraperitoneal (i.p.) injection at consecutive 5 days per week, for total 2 weeks. (G) The impact of IFIT5‐ or STAT1‐KD on the IFNγ‐induced miR‐128 or miR‐101 downregulation, compared to control vector (shCon). (H) The impact of mutant miR‐128 on the IFNγ‐facilitated sphere formation in DU145 cells. (ns = no significant differences, *p < .05, **p < .001, ***p < .0001)

Until now, the impact of IFN on PCSC is not well studied. As shown in Figure 5A, all three IFNs alone can increase prostasphere formation of sgTP53/RB1 LNCaP cells cultured with basal DMEM without B27, bFGF and EGF that are key ingredients to support the growth of prostasphere medium, suggesting IFNs are potent stimulatory factors in PCSC development. This phenomenon is further supported by that IFNγ exhibits a dose‐dependent stimulatory effect on the induction of CSC‐related genes (Figure 5B) as well as prostasphere formation (Figure S5A) in 22Rv1 cells. The similar impact of type I or type II IFN on prostasphere formation (Figure S5B) and upregulation of STAT1, IFIT5, BMI1, SOX2 and NANOG gene expression (Figure S5C) are also observed in DU145 cells. In contrast, fludarabine can diminish IFNβ‐induced DU145 prostasphere formation (Figure S5D).

Mounting evidence has demonstrated that CD44 is a critical regulator for self‐renewal, tumour initiation and metastasis in cancer stem cells. To further demonstrate the correlation between cancer stemness and IFN signalling activation, we isolated CD44^High^ and CD44^Low^ DU145 populations, and observed an increase in stemness properties along with higher level of JAK1, STAT1, IFIT5, BMI1, Nanog and SOX2 proteins expressing in CD44^High^ DU145 cells, when compared to CD44^Low^ DU145 subpopulation (Figure S5E). This evidence highly suggests the positive correlation between JAK‐STAT signalling and the self‐renewal capacity in PCSC subpopulation.

Knowing elevated STAT1 in sgTP53/RB1 LNCaP cells (Figure 1H), IFNγ treatment is expected to increase prostasphere formation under supplement‐free culture condition (Figure 5C) along with IFIT5 induction (Figure S5F). Furthermore, by knocking down STAT1 or IFIT5 in sgTP53/RB1 LNCaP cells, we observed significant reduction of the inductive effect of IFNγ on prostasphere formation (Figure 5D,E). Similarly, fludarabine exhibits therapeutic efficacy using in vivo tumour model of sgTP53/RB1 LNCaP (Left panel in Figure 5F), which also shows targeting efficiency (Right panel in Figure 5F). In contrast, both miR‐128 or miR‐101 level was rescued by either IFIT5 or STAT1 KD conditions in response to IFNγ treatment (Figure 5G). Functionally, elevated native miR‐128 or DSmut‐128 can antagonize IFNγ‐induced prostasphere formation in DU145 cells (Figure 5H), supporting the central role of STAT1‐IFIT5‐miR turnover in IFN‐induced PCSC.

Since SOX2 is the target of miR‐101, and BMI1 is found to be targeted by both miR‐101 and miR‐128 in PCa line (Figure 4F,G), we examined their roles in PCSC. Loss of BMI1 or SOX2 expression in DU145 cells significantly attenuates sphere formation and growth (Figure S5G). Meanwhile, SOX2 KD in the shTP53/RB1 LNCaP cells (Figure S5H) significantly diminished prostasphere formation (Figure S5I). Furthermore, BMI inhibitor‐PTC‐209 (Figure S5J) exhibit a dose‐dependent inhibition on prostasphere formation of two IFIT5‐high cell lines, DU145 (Figures S5K and S3G) and sgTP53/RB1‐LNCaP cells (Figures S5L and S3Q). Taken together, these data support the critical role of SOX2 or BMI1in PCSC activities.

### 3.7 STAT1 signalling activation by enzalutamide (ENZ) confers PCSC properties

**FIGURE 6:**
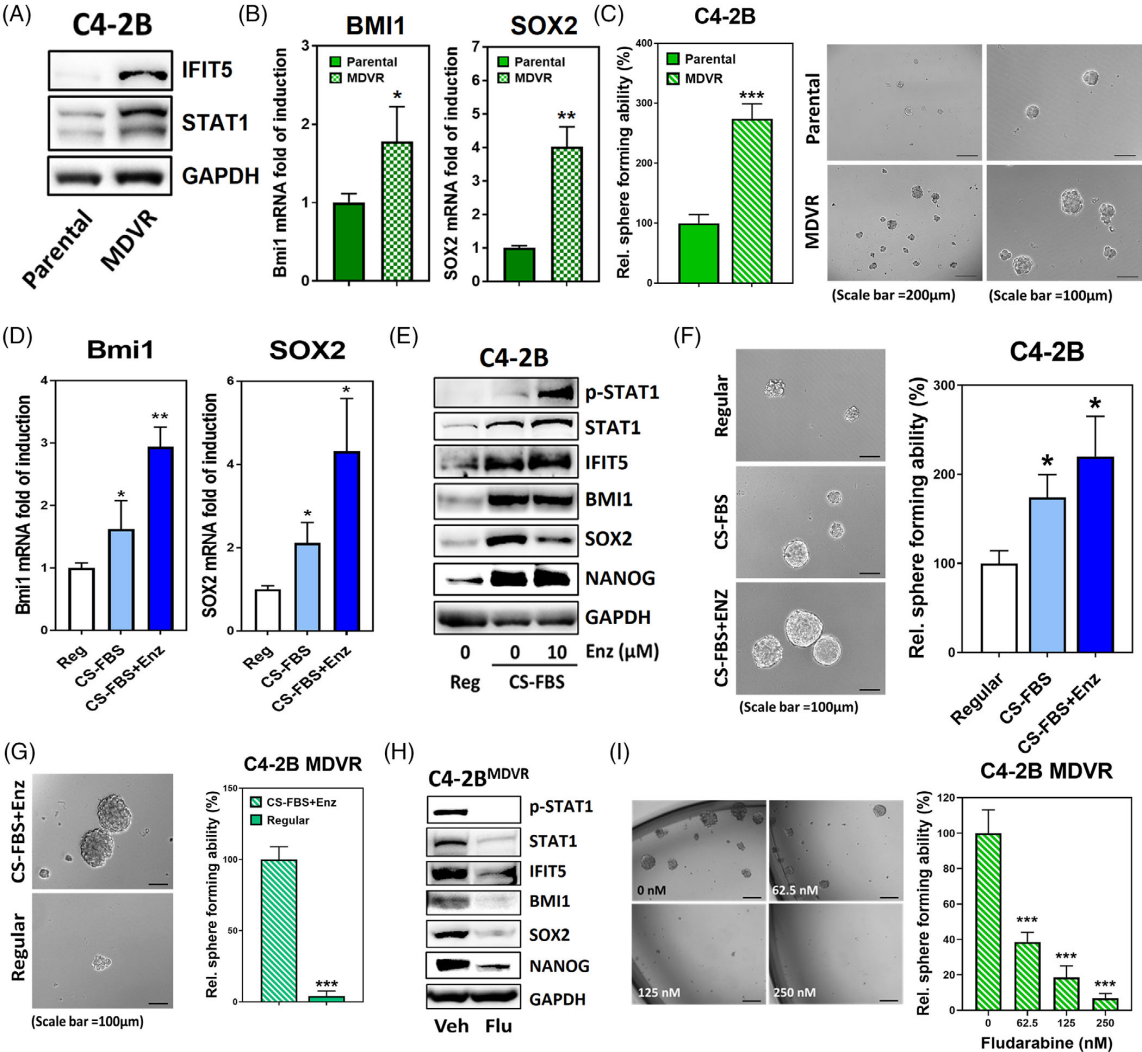
STAT1 signalling activation confers CSC properties in castration‐resistance PCa. (A) Elevation of STAT1 and IFIT5 protein in C4‐2B MDVR cells, compared to C4‐2B parental line. (B) Expression level of BMI1 and SOX2 mRNA in C4‐2B MDVR cells, compared to C4‐2B parental line. (C) Higher sphere forming ability of C4‐2B MDVR cells than C4‐2B parental cells. C4‐2B parental cells were cultured in 10% FBS RPMI‐1640 medium and C4‐2B MDVR cells were cultured in the Phenol Red free RPMI‐1640 medium supplemented with 10% CS‐FBS with additional 20 μM ENZ. (D) Induction of BMI1 and SOX2 gene upregulation in C4‐2B cells cultured in CS‐FBS‐supplemented phenol red‐free RPMI without (CS‐FBS) or with 10 μM ENZ (CS‐FBS+Enz), compared to regular culture condition (Reg). (E) Expression level of phosphorylated STAT1, STAT1, IFIT5, BMI1, SOX2 and NANOG proteins in C4‐2B cells cultured in CS‐FBS‐supplemented phenol red‐free RPMI without (CS‐FBS) or with 10 μM ENZ (CS‐FBS+Enz), compared to regular culture condition (Reg). (F) The sphere forming ability of C4‐2B cells primarily cultured in CS‐FBS‐supplemented pheno red‐free RPMI without (CS‐FBS) or with 10 μM ENZ (CS‐FBS+Enz) for 2 weeks, compared to regular culture condition (Reg). (G) The sphere forming ability of C4‐2B MDVR cells primarily cultured in regular condition for 2 weeks, compared with culture condition of CS‐FBS‐supplemented phenol red‐free RPMI with 20 μM ENZ (CS‐FBS+Enz). (H) The expression level of phosphorylated STAT1, STAT1, IFIT5, Bmi1, Sox2 and Nanog proteins in C4‐2B MDVR cells treated with fludarabine (500 nM, 48 h). (I) The sphere forming ability of C4‐2B MDVR cells treated with increased dose of fludarabine during sphere culture. C4‐2B MDVR cells were treated with fludarabine at corresponding concentration right after seeding into the ultra‐low attachment plate. Quantitation of spheres is done at 2 weeks after seeding. (*p < .05, **p < .001, ***p < .0001)

Accumulating evidence implies that ADT ultimately facilitates the lineage plasticity in PCa undergoing NE differentiation (NED). Noticeably, STAT1 and IFIT5 proteins (Figure 6A) as well as BMI1 and SOX2 mRNA (Figure 6B) are significantly elevated in the CRPC line, C4‐2B MDVR that is able to form more prostaspheres than parental cells (Figure 6C). To investigate whether the acquired stemness properties in CRPC cells is due to prolonged androgen‐deprived (AD) condition, we cultured the parental C4‐2B cells in 10% charcoal stripped‐FBS (CS‐FBS)—supplemented with Phenol Red free RPMI medium for 3 weeks. Although additional ENZ (10 μM) treatment could further enhance the AD‐induced BMI1 and SOX2 (Figure 6D) as well as STAT1 and IFIT5 mRNA levels (Figure S6A), it did not reflect on the protein expression from these genes (Figure 6E). On the other hand, following the upregulation of STAT1 and phosphorylated STAT1 (Figure 6E), a significant induction of IFNγ‐inducible STAT1‐driven genes are seen in C4‐2B cells cultured under AD condition with additional ENZ (Figure S6B). Nevertheless, additional ENZ treatment only slightly enhanced prostasphere formation of C4‐2B compared with AD condition alone (Figure 6F). Similar result is also observed in 22Rv1 cells (Figure S6C). In contrast, the prostasphere forming ability of castration‐resistant C4‐2B MDVR line is significantly abolished when the cells are switched to regular serum culture for 2 weeks, compared to AD condition (Figure 6G). Apparently, fludarabine is a potent agent in inhibiting PCSC activities from C4‐2B MDVR (Figure 6H,I), C4‐2B in AD (Figure S6D) or 22Rv1 cultured with ENZ (Figure S6E). The in vitro observation is also found in an in vivo sub‐cutaneous xenograft model. Both fludarabine and ruxolitinib treatments exhibit significant impact on the tumour growth of 22Rv1 line (Figure S6F,G), and diminish the protein level of STAT1, BMI1, SOX2 and NANOG in 22Rv1 tumours when compared with vehicle control (Figure S6H). On the other hand, administration of ENZ did not change much of the protein levels of either STAT1, BMI1, SOX2 or NANOG. Overall, these data support the central role of STAT1‐IFIT5 pathway in ENZ‐elicited PCSC.

## 4 DISCUSSION

PCa is a typical androgen‐dependent disease. Thus ADT is considered the most effective regimen to treat metastatic hormone naïve PCa; almost all patients eventually develop t‐CRPC that is associated with the mortality of this disease. Although many new agents have been introduced for these patients, inevitably, CRPC acquires resistance to become t‐CRPC, which is considered as a lethal disease without effective targeted therapy. Approximately, 20%–25% t‐CRPC patients recurred from ADT harbour neuroendocrine cell features from classical adenocarcinoma PCa (ADPC), also, these cells exhibit ADT‐ or radio‐resistance. Thus, understanding the molecular driver(s) leading to the onset of NEPC can certainly lead us to develop new therapeutic strategies for the recurrent t‐CRPC.

In general, ADPC cells exhibit differentiated luminal cell markers. In contrast, NEPC cells express neuronal biomarkers and neuronal factors secretion in an endocrine fashion. The molecular characterization of PCa neuroendocrine differentiation has been thoroughly studied among PCa patients with NE‐like or ADPC tumour feature. Among the genes that upregulated in NE‐like tumour specimens when compared to ADPC, many are either neuroendocrine markers, neuropeptides, or castration‐resistance‐related genes.^27^

Based on distinct phenotypes among ADPC and NEPC, lineage plasticity is likely manifested as reversible or irreversible changes in cellular ‘identity’, whereby cells take on an alternative morphologic, phenotypic, or epigenetic state. Mechanisms empowering the lineage plasticity in PCa are also critical for the onset of NED in t‐CRPC patients. Loss of function in Rb and TP53 gene^3^, ^28^ or gene amplification of N‐MYC^29^, ^30^ or Aurora A kinase^31^, ^32^ is considered as key genetic predisposition to PCSC associated with elevated SOX2 or BMI1 expression; SOX2 has been shown to upregulate BRN2 and synaptophysin (SYP) leading to NEPC^33^ and BMI1^+^SOX2^+^ cells drive the recurrent PCa.^34^ In addition to genetic alteration, it appears that ADT can facilitate the lineage plasticity of PCa, indicating an important role of epigenetic factors in facilitating PCSC development.

Previously, we demonstrated the promoting role of IFN in EMT of PCa^14^ since significant elevation of IFN levels are found in the serum of PCa patients receiving ADT.^35^ Furthermore, IRDS comprised a subset of IFN‐induced genes that are highly associated with therapeutic resistance to chemotherapy.^13^ A more recent analysis of TCGA PCa dataset indicated that elevated IRDS is correlated with decreased disease‐free survival in PCa patients.^12^ Meanwhile, a study focused on germline variation suggested that IRDS is more prevalent in the tumour specimens derived from African American PCa patients with higher incidence and mortality rate, compared to European American cohorts.^12^, ^36^ Taken together, IFN‐elicited signalling pathway is highly involved in PCa progression.

STAT1 or STAT2 are essential components in the IFN‐induced signalling pathway that leads to transcriptional activation of IFN‐stimulated genes (ISGs). In our observation, STAT1 and several STAT1‐driven IRDS genes are highly upregulated not only in the cohort of PCa patients with lymph node metastasis (Figure 1D) but also in several PCa lines with acquired lineage plasticity (Figure 1H). Noticeably, we found STAT1 signalling is highly activated in both sgTP53/RB1‐LNCaP and androgen‐deprived C4‐2B lines with enhanced PCSC properties and increased sphere forming abilities (Figures 1G,H and 6A,C). Meanwhile, a significant enrichment of IFN responsive genes and activation of STAT1 signalling are also observed in ARCaP subline with NEPC features (Figure 2E). Functionally, the upregulation of STAT1 and STAT1‐driven genes in several CRPC lines lead to enhanced PCSC capabilities (Figure S6B and Figure 6E,F) that can provide pro‐survival advantage of ADPC under ADT as well as facilitate EMT underlying metastasis or recurrent cells with NE phenotypes. Similarly, IL6‐elicited STAT3 activation is associated with PCa metastasis, ENZ resistance, and NED.^37^, ^38^, ^39^, ^40^ Altogether, activated JAK‐STAT pathways play a master driver role of PCa progression and lineage plasticity.

Mechanistically, IFIT5, an IFN‐induced protein with tetratricopeptide repeats that can bind to 5′end viral RNA and cause its degradation, plays a key downstream effector in modulating stemness gene expression (Figure S3L,P). Particularly, we demonstrated that loss of IFIT5 in PCa lines leads to attenuated stemness properties and clonogenicity in vitro (Figure 3G and Figure S3I), and impacts on the tumorigenicity in vivo (Figure 3H). In addition to its functional role in viral RNA degradation, the unique function of IFIT5 in PCa cells is to modulate the turnover of miR subpopulation via recognizing the 5′end specific structure of precursor form (Figure 4F,G); similar observations were found in bladder cancer.^41^ Noticeably, either miR‐101 or ‐128, the target of IFIT5, can degrade SOX2 or BMI1 mRNA leading to the suppression of PCSC activities (Figure 4B–E). Particularly, the role of miR‐128 on targeting BMI1 is also demonstrated by the study done by Jin M. et al. They observed that cell population with lower miR‐128 level possessed higher clonogenicity and tumorigenic activities when compared to miR‐128‐high population.^42^ By targeting STAT1 or JAK1 activation with specific inhibitors such as fludarabine or ruxolitinib, our data clearly demonstrated that JAK1‐STAT1 signalling pathway is a critical pathway associated with PCSC and NED (Figures 1J and 2G; Figures S1G and S2E). Also, fludarabine, FDA‐approved chronic lymphocytic leukaemia therapeutic, exhibited a potent efficacy in suppressing t‐CRPC tumour (Figures 2H and 5F). This result supports the re‐purposing of this agent on t‐CRPC treatment, which is expected to have an immediate impact on overall survival of PCa patients.

## 5 CONCLUSION

Although several new agents such as PARP inhibitor and Lu^177^‐PSMA show some promising clinical efficacy for t‐CRPC, this disease is by far the most lethal urologic malignancy and the molecular cellular mechanism is not fully understood. The activation of JAK1‐STAT1‐IFIT5 pathway plays a critical role in CSC phenotypes leading to t‐CRPC. Thus, targeting JAK or STAT1 using FDA‐approved specific small molecule inhibitors (SMIs) can significantly inhibit CSC phenotypes both in vitro and in vivo models, which offer new therapeutic regimens to prolong overall survival of these patients.

## CONFLICT OF INTEREST

The authors declare that there is no conflict of interest that could be perceived as prejudicing the impartiality of the research reported.

## CONSENT FOR PUBLICATION

All the authors have agreed with the content of this manuscript.

## Supporting information

Supporting Information
