# Supplementary material for: The driver role of JAK‐STAT signalling in cancer stemness capabilities leading to new therapeutic strategies for therapy‐ and castration‐resistant prostate cancer
Source: Clin Transl Med. 2022 Jul 31;12(8):e978. doi: 10.1002/ctm2.978 (PMC9339240; doi:10.1002/ctm2.978)
Supplement: Supplementary file 1 — Supporting Information [file CTM2-12-e978-s001.pdf]

# SUPPLEMENTARY FIGURES AND TABLE

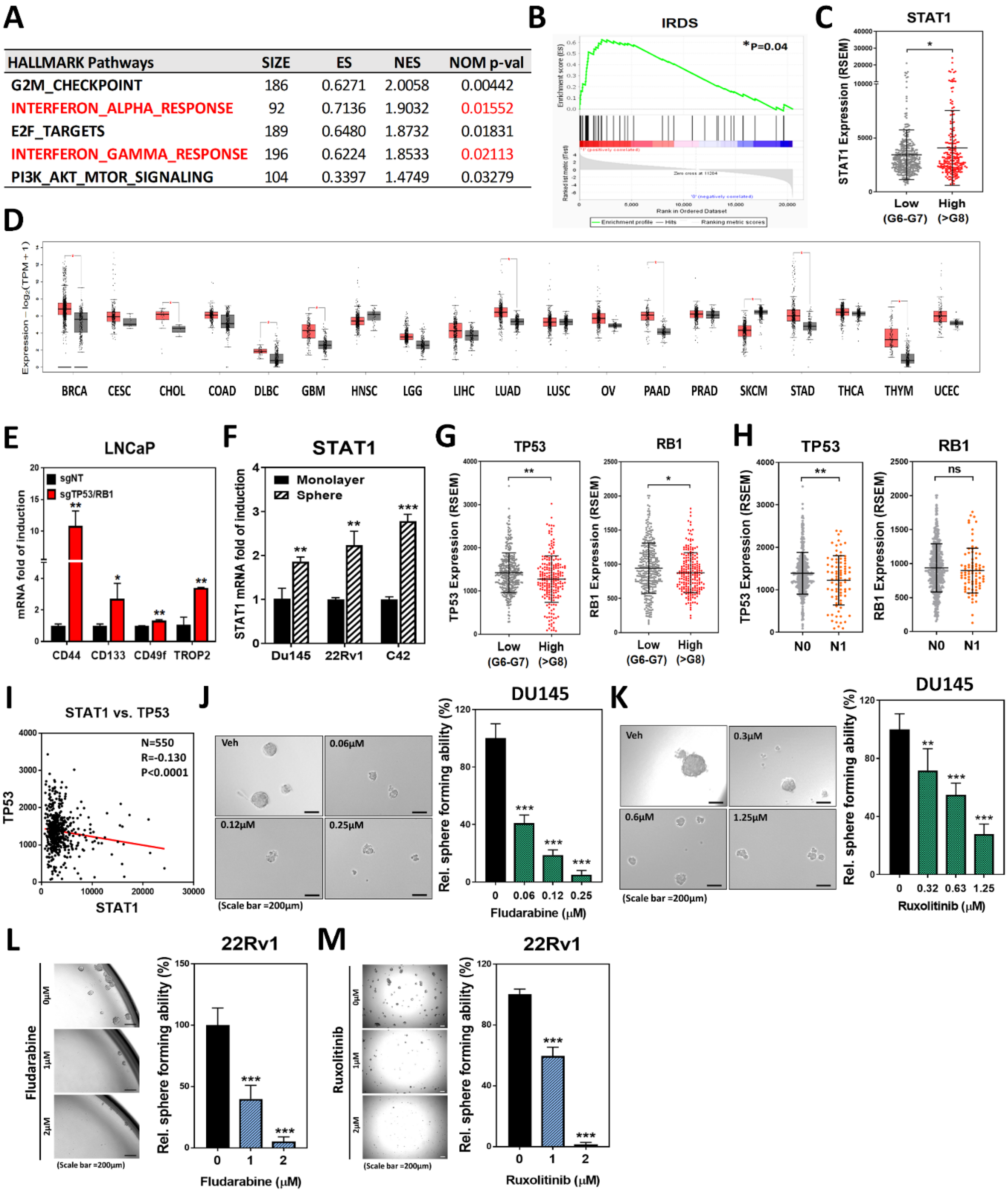

**Figure S1.** (**A**) GSEA identifying enrichment of upregulated genes associated with each specific pathway in PCa patients with lymph node metastasis (N1, n=80), compared to cohort without metastasis (N0, n=345). (**B**) GSEA identifying enrichment of IRDS genes in the tumor specimens of PCa patients with lymph node metastasis (N1, n=80), compared to cohort without metastasis (N0, n=345). (**C**) TCGA PCa dataset demonstrating STAT1 expression level among low (Gleason score=6 and 7, n=292) and High (Gleason score ≥8, n=206) grade tumor specimens from PCa patients. (**D**) GEPIA analysis of STAT1 expression level compared among tumor (red) and normal (grey) specimens derived from different malignancies (BRCA, Breast invasive adenocarcinoma [ca]; CESC, Cervical endocervical squamous cell ca; CHOL, Cholangio ca; COAD, Colon ca; DLBC, Diffuse large B-cell lymphoma; GBM, glioblastoma multiforme; HNSC, Head-neck squamous ca; LGG, Low-grade gliomas; LIHC, Liver hepatocellular ca; LUAD, Lung ca; LUSC, Lung squamous cell ca; OV, Ovarian serous cystadenocarcinoma; PAAD, Pancreatic ca; PRAD, Prostate ca; SKCM, Skin cutaneous melanoma; STAD, Stomach ca; THCA, Thyroid ca; THYM, Thymoma; UCEC, Uterine corpus endometrial ca) (*p<0.05 indicated significant differences between tumor vs. normal benign tissue) (http://gepia.cancer-pku.cn/index.html). (**E**) Expression level of cancer stemness-associated genes (CD44, CD133, CD49f and TROP2) in sgTP53/RB1-LNCaP cell, compared to control vector (sgNT). (**F**) Induction of STAT1 mRNA upregulation in the sphere (S) derived from each PCa line, compared to corresponding monolayer (M) culture. (**G**) TCGA PCa dataset demonstrating TP53 and RB1 expression level among low (Gleason score=6 and 7, n=292) and High (Gleason score ≥8, n=206) grade tumor specimens from PCa patients. (**H**) TCGA PCa dataset demonstrating expression of TP53 and RB1 in PCa patients with lymph node metastasis (N1, n=80), compared to cohort without metastasis (N0, n=345). (**I**) Clinical correlation of STAT1 with TP53 expression (N=550) based on TCGA PCa dataset analysis. (**J, K**) Dose-dependent impact of Fludarabine and Ruxolitinib on the sphere forming ability of DU145 cells. (**L, M**) Dose-dependent impact of Fludarabine or Ruxolitinib on the sphere forming ability of 22Rv1 cells. Du145 or 22Rv1 cells were treated with either Fludarabine or Ruxolitinib at corresponding concentration right after seeding into the ultra-low attachment plate. Quantitation of sphere forming ability is done at 2 weeks after seeding (ns=no significant differences, *p<0.05, **p<0.001, ***p<0.0001)

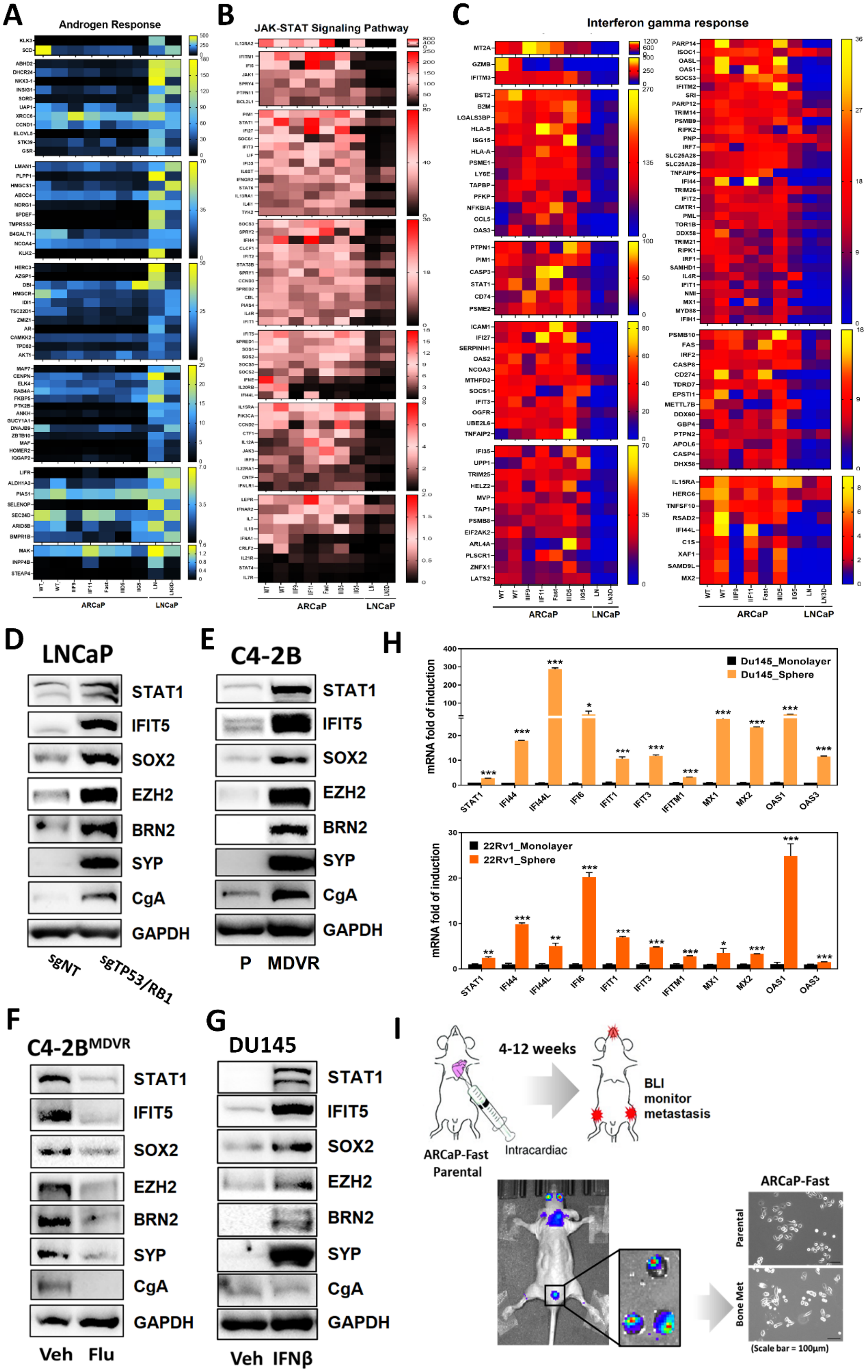
A
Androgen Response
ARCaP
LNCaP
B
JAK-STAT Signaling Pathway
ARCaP
LNCaP
C
Interferon gamma response
ARCaP
LNCaP
D
LNCaP
STAT1
IFIT5
SOX2
EZH2
BRN2
SYP
CgA
GAPDH
SgNT
SgTP53/RB1
E
C4-2B
STAT1
IFIT5
SOX2
EZH2
BRN2
SYP
CgA
GAPDH
P
MDVR
H
Du145_Monolayer
Du145_Sphere
mRNA fold of induction
STAT1
IFI44
IFI44L
IFI6
IFIT1
IFIT3
IFITM1
MX1
MX2
OAS1
OAS3
22Rv1_Monolayer
22Rv1_Sphere
mRNA fold of induction
F
C4-2B[MDVR]
STAT1
IFIT5
SOX2
EZH2
BRN2
SYP
CgA
GAPDH
Veh
Flu
G
DU145
STAT1
IFIT5
SOX2
EZH2
BRN2
SYP
CgA
GAPDH
Veh
IFNβ
I
4-12 weeks
BLI
monitor
metastasis
ARCaP-Fast
Parental
Intracardiac
ARCaP-Fast
Parental
Bone Met
(Scale bar = 100μm)

**Figure S2.** (**A**) Heat map illustrating the Androgen Response gene expression profile among ARCaP sublines (From Left to Right: ARCaP-WT-1, ARCaP-WT-2, ARCaP-IIIF9, ARCaP-IIF11, ARCaP-Fast, ARCaP-IIID5, and ARCaP-IIG5) LNCaP and LNCaP-3D line. (**B**) Heat map illustrating the JAK-STAT signaling pathway gene expression profile among ARCaP sublines (From Left to Right: ARCaP-WT-1, ARCaP-WT-2, ARCaP-IIIF9, ARCaP-IIF11, ARCaP-Fast, ARCaP-IIID5, and ARCaP-IIG5), LNCaP and LNCaP-3D line. (**C**) Heat map illustrating the IFNγ-responsive gene expression profile among ARCaP sublines (From Left to Right: ARCaP-WT-1, ARCaP-WT-2, ARCaP-IIIF9, ARCaP-IIF11, ARCaP-Fast, ARCaP-IIID5, and ARCaP-IIG5), LNCaP and LNCaP-3D line. (**D**) Protein expression level of STAT1, IFIT5, SOX2, EZH2, BRN2, SYP, and CgA in sgTP53/RB1-LNCaP cell, compared to sgNT control. (**E**) Expression level of STAT1, IFIT5, SOX2, EZH2, BRN2, SYP and CgA proteins among C4-2B parental and MDVR lines. (**F**) Downregulation of STAT1, IFIT5, SOX2, EZH2, BRN2, SYP and CgA proteins in C4-2B MDVR line treated with Fludarabine (500 nM, 48 hrs). (**G**) Induction of STAT1, IFIT5, SOX2, EZH2, BRN2, SYP and CgA proteins in DU145 cells treated with IFNβ (20 ng/ml, 48 hrs). (**H**) Expression of ten IFN-inducible STAT1-driven genes in DU145 and 22Rv1 tumor spheres, compared to corresponding monolayer adherent culture. (**I**) Intra-cardiac injection model to establish bone metastatic subline of ARCaP-Fast. (ns=no significant differences, *$p<0.05$, **$p<0.001$, ***$p<0.0001$)

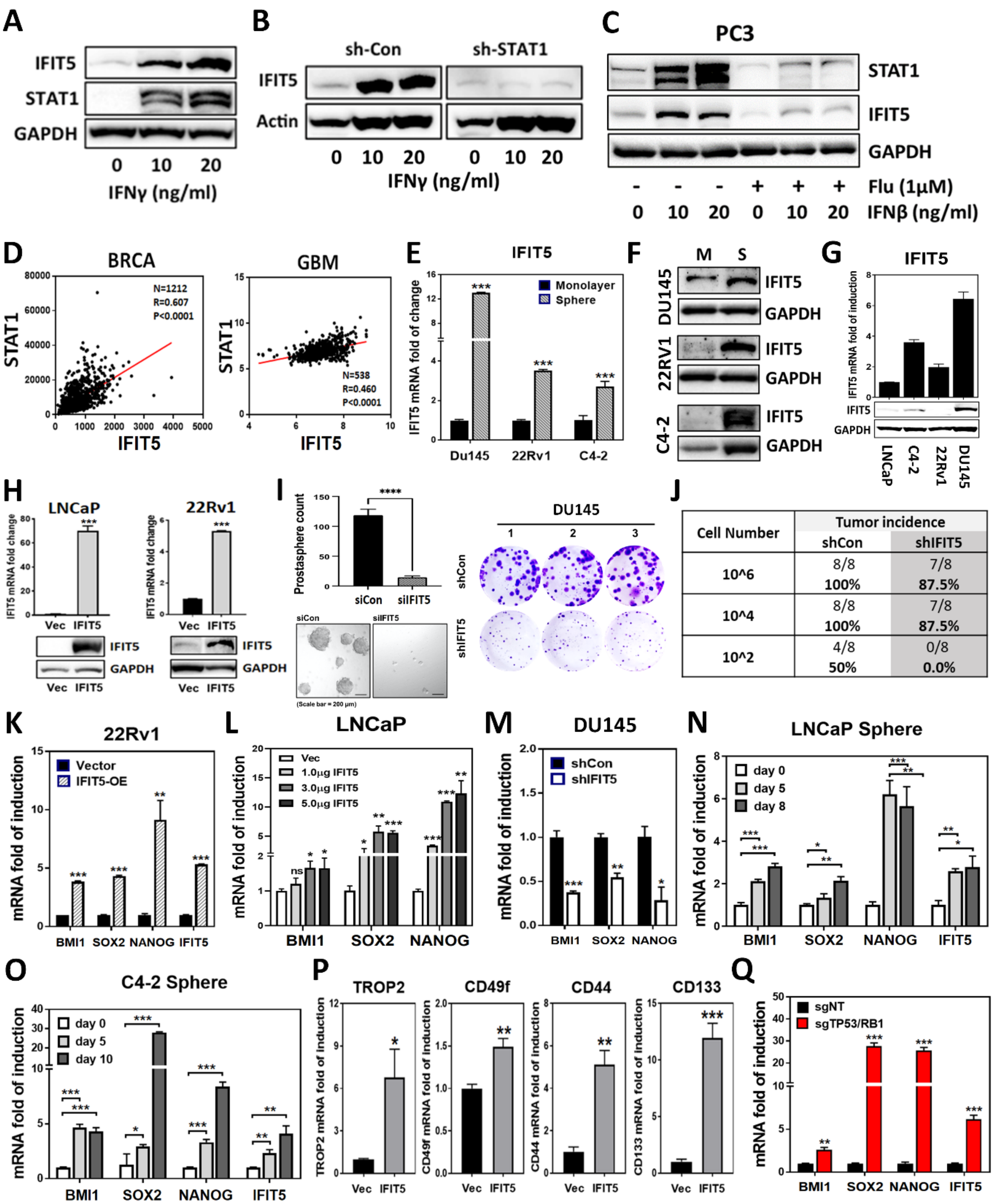
A
IFIT5
STAT1
GAPDH
0 10 20
IFNγ (ng/ml)
B
sh-Con
sh-STAT1
IFIT5
Actin
0 10 20 0 10 20
IFNγ (ng/ml)
C
PC3
STAT1
IFIT5
GAPDH
- - - + + + Flu (1μM)
0 10 20 0 10 20 IFNβ (ng/ml)
D
BRCA
N=1212
R=0.607
P<0.0001
STAT1
IFIT5
GBM
N=538
R=0.460
P<0.0001
E
IFIT5
Monolayer
Sphere
IFIT5 mRNA fold of change
Du145 22Rv1 C4-2
F
M S
DU145
22RV1
C4-2
IFIT5
GAPDH
G
IFIT5
IFIT5 mRNA fold of induction
LNCaP C4-2 22Rv1 DU145
H
LNCaP
22Rv1
IFIT5 mRNA fold change
Vec IFIT5
I
Prostasphere count
siCon siIFIT5
(Scale bar = 200 μm)
DU145
1 2 3
shCon
shIFIT5
J
Cell Number
Tumor incidence
shCon
shIFIT5
10^6 8/8 100% 7/8 87.5%
10^4 8/8 100% 7/8 87.5%
10^2 4/8 50% 0/8 0.0%
K
22Rv1
Vector
IFIT5-OE
mRNA fold of induction
BMI1 SOX2 NANOG IFIT5
L
LNCaP
Vec
1.0μg IFIT5
3.0μg IFIT5
5.0μg IFIT5
M
DU145
shCon
shIFIT5
N
LNCaP Sphere
day 0
day 5
day 8
O
C4-2 Sphere
day 0
day 5
day 10
P
TROP2
CD49f
CD44
CD133
Vec IFIT5
Q
sgNT
sgTP53/RB1
BMI1 SOX2 NANOG IFIT5

**Figure S3.** (**A**) IFNγ induced upregulation of IFIT5 protein and mRNA in DU145 cells. (**B**) Impact of STAT1 shRNA knockdown on IFNγ-induced upregulation of IFIT5 protein in DU145 cells. (**C**) Impact of Fludarabine-mediated STAT1 inhibition on IFNγ-induced upregulation of IFIT5 protein in PC3 cells. Cells were treated with IFN for 48hrs before harvest for western blot analysis. (**D**) TCGA dataset demonstrating the clinical correlation of IFIT5 with STAT1 in Breast invasive carcinoma (BRCA, N=1212) and Glioblastoma Multiform (GBM, N=538). (**E**) Upregulation of IFIT5 mRNA level in the sphere (S) derived from each PCa line, compared to corresponding monolayer (M) culture. (F) IFIT5 protein upregulation in the sphere (S) derived from each PCa line, compared to corresponding monolayer (M) culture. (**G**) The profile of IFIT5 mRNA and protein expression among PCa cell lines was normalized with LNCaP cell line. (**H**) Level of IFIT5 mRNA and protein in IFIT5-overexpressing (IFIT5) LNCaP and 22Rv1 cells, compared to vector control (Vec). (**I**) The impact of IFIT5 siRNA on the sphere forming ability and clonogenicity of DU145 cells. (**J**) The tumor incidence of subcutaneous injected IFIT5-knockdown (shIFIT5) DU145 cells at $10^6$, $10^4$ and $10^2$ cell number, compared to shCon cohort. (**K**) Expression level of Bmi1, Sox2, Nanog and IFIT5 in IFIT5-overexpressed 22Rv1 cells, compared to vector control. (**L**) Dose-dependent upregulation of BMI1, SOX2, NANOG in IFIT5-overexpressed LNCaP cells, compared to vector control. (**M**) Expression level of BMI1, SOX2 and Nanog in IFIT5-knockdown DU145 cells was normalized with the control vector (shCon). (**N, O**) Induction of BMI1, SOX2, Nanog and IFIT5 gene upregulation during LNCaP and C4-2 tumor sphere formation. (**P**) Expression level of TROP2, CD49f, CD44 and CD133 mRNA in IFIT5-overexpressing LNCaP cells compared to vector control (Vec). (**Q**) mRNA upregulation of BMI1, SOX2, NANOG and IFIT5 in sgTP53/RB1-LNCaP cell, compared to control vector (sgNT). (ns=no significant differences, *$p<0.05$, **$p<0.001$, ***$p<0.0001$)

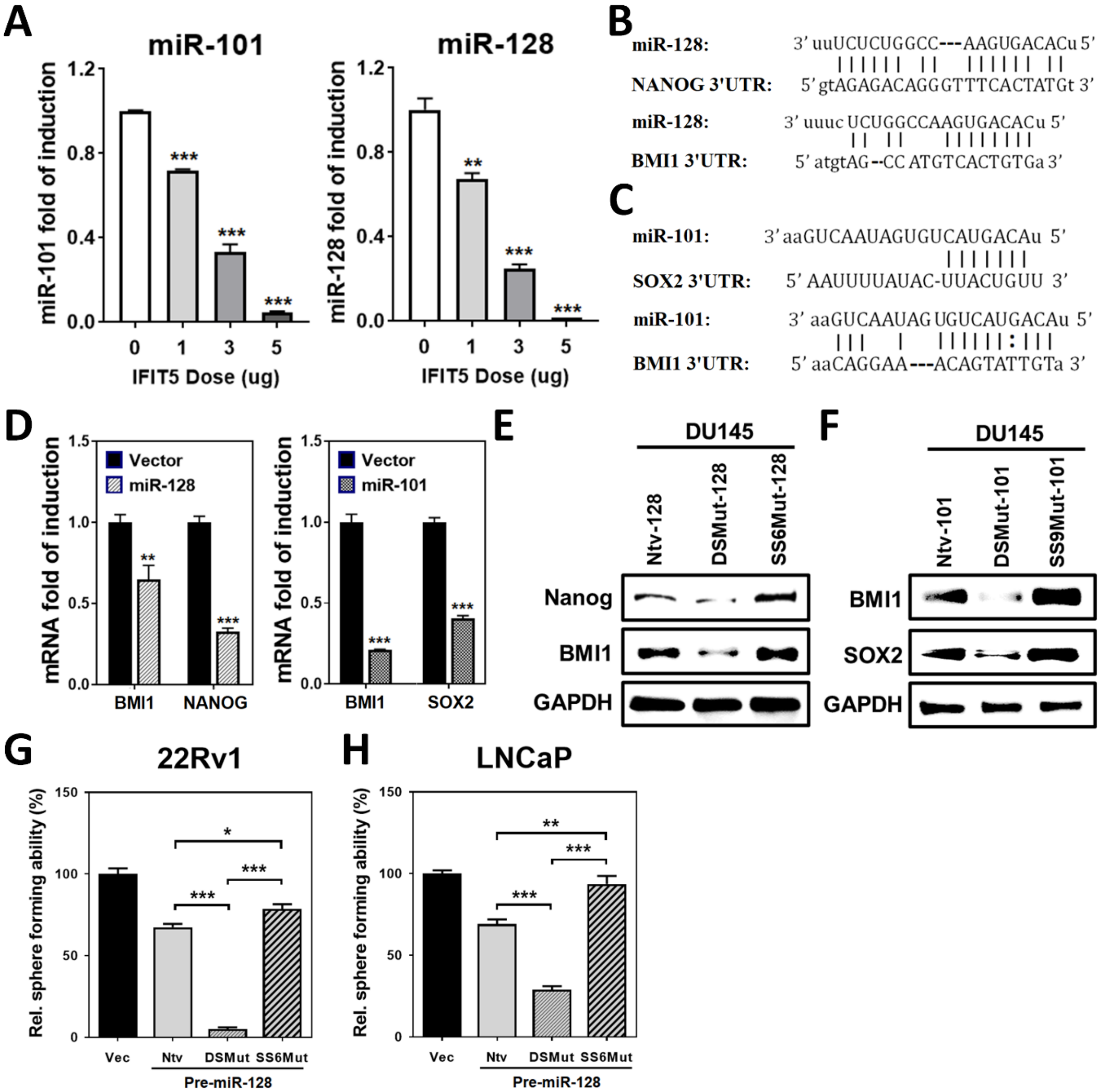
A
miR-101
miR-101 fold of induction
IFIT5 Dose (ug)
miR-128
miR-128 fold of induction
IFIT5 Dose (ug)
B
miR-128: 3' uuUCUCUGGCC---AAGUGACACu 5'
NANOG 3'UTR: 5' gtAGAGACAGGGTTTCACTATGt 3'
miR-128: 3' uuuc UCUGGCCAAGUGACACu 5'
BMI1 3'UTR: 5' atgtAG--CC ATGTCACTGTGa 3'
C
miR-101: 3' aaGUCAAUAGUGUCAUGACAu 5'
SOX2 3'UTR: 5' AAUUUUAUAC-UUACUGUU 3'
miR-101: 3' aaGUCAAUAG UGUCAUGACAu 5'
BMI1 3'UTR: 5' aaCAGGAA---ACAGTATTGTa 3'
D
Vector
miR-128
mRNA fold of induction
BMI1
NANOG
Vector
miR-101
BMI1
SOX2
E
DU145
Ntv-128
DSMut-128
SS6Mut-128
Nanog
BMI1
GAPDH
F
DU145
Ntv-101
DSMut-101
SS9Mut-101
BMI1
SOX2
GAPDH
G
22Rv1
Rel. sphere forming ability (%)
Vec
Ntv
DSMut
SS6Mut
Pre-miR-128
H
LNCaP
Rel. sphere forming ability (%)
Vec
Ntv
DSMut
SS6Mut
Pre-miR-128

**Figure S4.** (**A**) Dose-dependent impact of IFIT5-OE on the level of miR-101 and miR-128 expression in 22Rv1 cells. Cells were harvested for RNA extraction and qRT-PCT analysis of miRNA expression at 48 hrs after transfection. (**B**) Representative matched nucleotide sequences between the seed region of miR-128 and the 3'UTR of NANOG and BMI1 mRNA. (**C**) Representative matched nucleotide sequences between the seed region of miR-101 and the 3'UTR of SOX2 and BMI1 mRNA (**D**) Left: The impact of miR-128 OE on BMI1 and NANOG level in 22Rv1 cells. Right: The impact of miR-101 OE on BMI1 and SOX2 level in 22Rv1 cells. (**E, F**) BMI1, SOX2 or NANOG protein expression in DU145 cells transfected with pre-miR-128 or pre-miR-101 mutant constructs. Cells were subjected to western blot analysis 48 hrs after transfection. (**G, H**) The impact of Native, DSMut or SS6Mut pre-miR-128 on the sphere formation of 22Rv1 and LNCaP cells. Cells were seeding to the ultra-low attachment plate 48hrs after transfection (* $p<0.05$, ** $p<0.001$, *** $p<0.0001$)

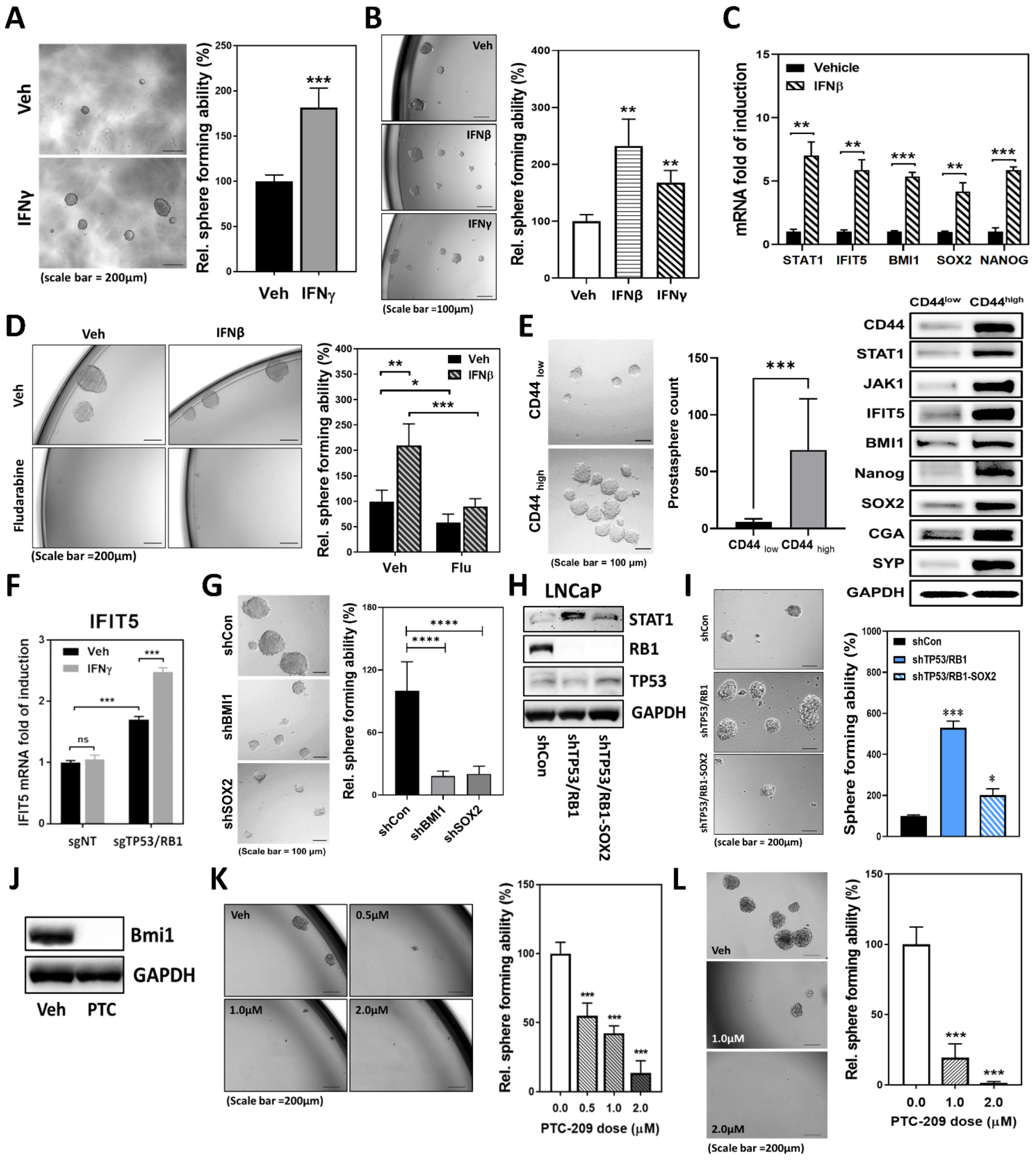

A
Veh
IFNγ
(scale bar = 200μm)
Rel. sphere forming ability (%)
Veh
IFNγ
***
B
Veh
IFNβ
IFNγ
(Scale bar =100μm)
Rel. sphere forming ability (%)
C
Vehicle
IFNβ
mRNA fold of induction
STAT1
IFIT5
BMI1
SOX2
NANOG
D
Veh
IFNβ
Fludarabine
(Scale bar =200μm)
Rel. sphere forming ability (%)
Flu
E
CD44 low
CD44 high
(Scale bar = 100 μm)
Prostasphere count
CD44low
CD44high
CD44
STAT1
JAK1
IFIT5
BMI1
Nanog
SOX2
CGA
SYP
GAPDH
F
IFIT5
IFIT5 mRNA fold of induction
ns
sgNT
sgTP53/RB1
G
shCon
shBMI1
shSOX2
(Scale bar = 100 μm)
Rel. sphere forming ability (%)
H
LNCaP
STAT1
RB1
TP53
GAPDH
shCon
shTP53/RB1
shTP53/RB1-SOX2
I
(scale bar = 200μm)
Sphere forming ability (%)
J
Bmi1
GAPDH
Veh
PTC
K
0.5μM
1.0μM
2.0μM
(Scale bar =200μm)
Rel. sphere forming ability (%)
PTC-209 dose (μM)
L
(Scale bar =200μm)
Rel. sphere forming ability (%)
PTC-209 dose (μM)

**Figure S5.** (**A**) The impact of IFNγ on 22Rv1 tumor sphere formation. (**B**) The impact of IFNβ and IFNγ on the sphere formation of DU145 cells (**C**) IFNβ-induced upregulation of STAT1, IFIT5, Bmi1, Sox2 and Nanog in DU145 cells. (**D**) The impact of Fludarabine on the IFNβ-facilitated sphere forming ability of DU145 cells. (**E**) Left: Comparison of sphere forming ability between $CD44^{High}$ and $CD44^{Low}$ DU145 subpopulation. Right: Differential protein expression level of CD44, STAT1, JAK1, IFIT5, BMI1, NANOG, SOX2, CgA and SYP between CD44High and $CD44^{Low}$ DU145 subpopulation. (**F**) The impact of IFNγ on the induction of IFIT5 in sgTP53/RB1-LNCaP cells, compared to sgNT control. (**G**) The impact of BMI1 or SOX2 knockdown on the DU145 sphere formation and growth. (**H**) Protein level of STAT1, RB1 and TP53 in shTP3/RB1 vs. shTP3/RB1/SOX2 LNCaP cells. (**I**) The sphere forming ability of shTP3/RB1 vs. shTP3/RB1/SOX2 LNCaP cells. (**J**) Suppression of BMI1 protein level in DU145 cells treated with BMI1 inhibitor PTC-209 (500 nM, 48 hrs). (**K, L**) Dose-dependent suppression on the sphere formation of DU145 and sgTP53/RB1-LNCaP cells treated with PTC-209. (ns=no significant differences, *p<0.05, **p<0.001, ***p<0.0001)

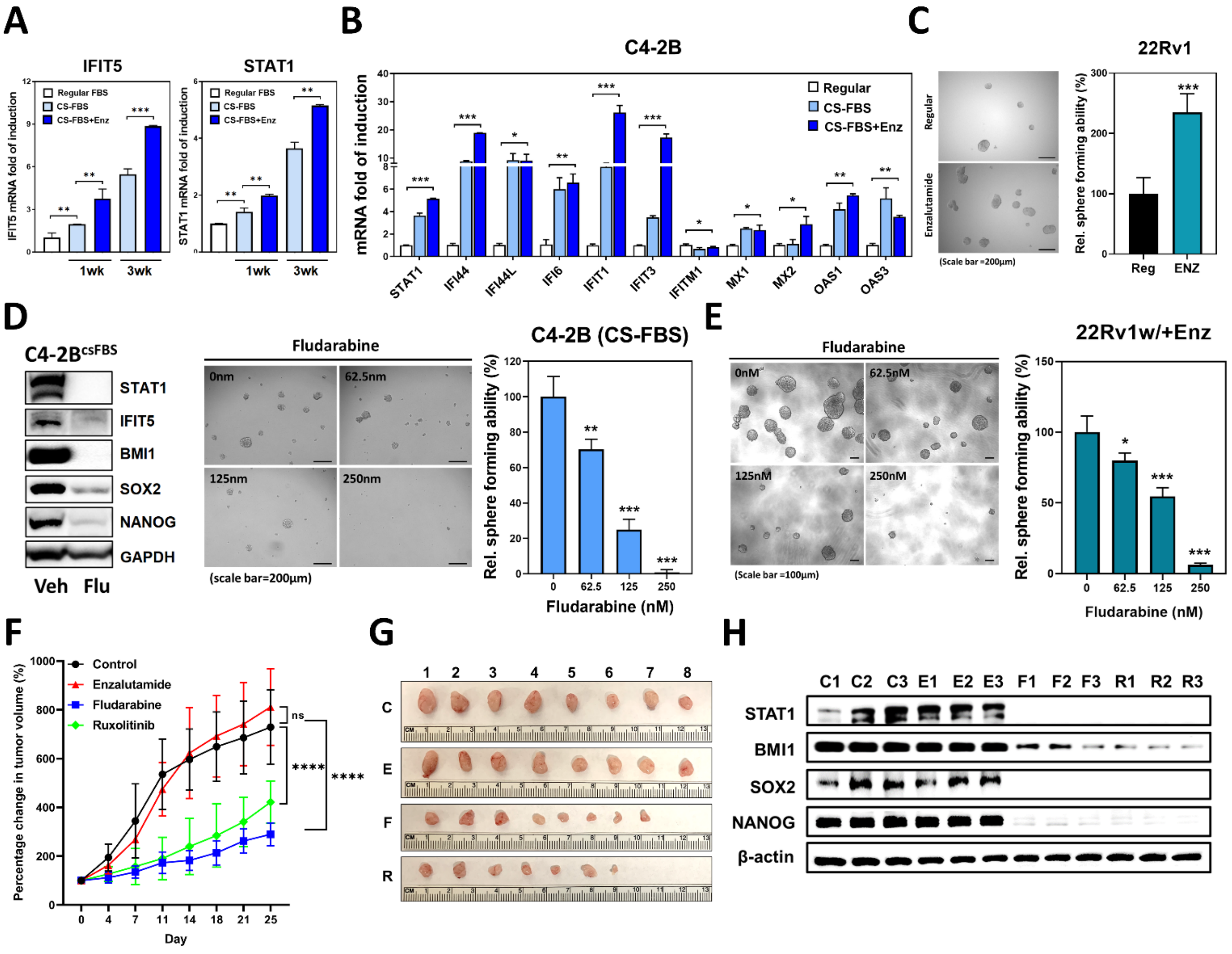
A
IFIT5
STAT1
IFIT5 mRNA fold of induction
STAT1 mRNA fold of induction
Regular FBS
CS-FBS
CS-FBS+Enz
1wk
3wk
B
C4-2B
mRNA fold of induction
Regular
CS-FBS
CS-FBS+Enz
STAT1
IFI44
IFI44L
IFI6
IFIT1
IFIT3
IFITM1
MX1
MX2
OAS1
OAS3
C
22Rv1
Regular
Enzalutamide
(Scale bar =200μm)
Rel. sphere forming ability (%)
Reg
ENZ
D
C4-2BcsFBS
STAT1
IFIT5
BMI1
SOX2
NANOG
GAPDH
Veh
Flu
Fludarabine
0nm
62.5nm
125nm
250nm
(scale bar=200μm)
C4-2B (CS-FBS)
Rel. sphere forming ability (%)
Fludarabine (nM)
E
Fludarabine
0nM
62.5nM
125nM
250nM
(Scale bar =100μm)
22Rv1w/+Enz
Rel. sphere forming ability (%)
Fludarabine (nM)
F
Control
Enzalutamide
Fludarabine
Ruxolitinib
Percentage change in tumor volume (%)
Day
ns
****
****
G
1 2 3 4 5 6 7 8
C
E
F
R
H
C1 C2 C3 E1 E2 E3 F1 F2 F3 R1 R2 R3
STAT1
BMI1
SOX2
NANOG
β-actin

**Figure S6.** (**A**) Time-dependent elevation of STAT1 or IFIT5 mRNA level in C4-2B cells cultured in CS-FBS-supplemented phenol red-free RPMI without or with additional 10 μM ENZ. (**B**) Induction of ten IFN-inducible STAT1-driven genes in C4-2B primarily cultured with CS-FBS without or with additional ENZ (10 μM), compared to regular medium. (**C**) The sphere forming ability of 22Rv1 cells cultured in androgen-deprived condition (5 μM ENZ) or regular condition (Reg). (**D**) Left: The impact of Fludarabine treatment (500 nM, 48 hrs) on the expression level of STAT1, IFIT5, BMI1, SOX2 and NANOG proteins in C4-2B cells cultured in CS-FBS-supplemented phenol red-free RPMI for 3 weeks. Middle-Right: The dose-dependent impact of Fludarabine on the sphere forming ability of C4-2B cells primarily cultured in CS-FBS-supplemented phenol red-free RPMI for 2 weeks. (**E**) The dose-dependent impact of Fludarabine on the sphere forming ability of 22Rv1 cells primarily cultured in androgen-deprived condition with 5 μM ENZ for 2 weeks. (**F**) The effect of ENZ (20 mg/kg), Fludarabine (20 mg/kg) and Ruxolitinib (20 mg/kg) on 22Rv1 tumor growth. (**G**) The tumor size of vehicle control (C), ENZ (E), Fludarabine (F) and Ruxolitinib (R) treatment groups. (**H**) Expression of STAT1, BMI1, SOX2 and NANOG protein levels in the 22Rv1 tumors treated with ENZ (E), Fludarabine (F) and Ruxolitinib (R), and vehicle control (C). All drugs were administrated through i.p. injection 5 days per week for 2 weeks. (****$p<0.0001$)

## SUPPLEMENTARY TABLEs

**Supplementary Table 1. GSEA analyses of pathways representing genes overexpression in ARCaP-IIF11 compared to ARCaP-Fast and ARCaP-IIB5 sublines.**

| NAME | NOM p-val | SIZE | ES | NES | FDR q-val |
|---|---|---|---|---|---|
| HALLMARK_INTERFERON_GAMMA_RESPONSE | <0.0001 | 197 | 0.902104 | 1.113394 | 0.9031964 |
| HALLMARK_INTERFERON_ALPHA_RESPONSE | <0.0001 | 94 | 0.941871 | 1.106214 | 0.8066409 |
| HALLMARK_PI3K_AKT_MTOR_SIGNALING | <0.0001 | 104 | 0.803492 | 1.211047 | 1 |
| HALLMARK_KRAS_SIGNALING_DN | <0.0001 | 194 | 0.779206 | 1.186822 | 1 |
| HALLMARK_HEDGEHOG_SIGNALING | 0.22166666 | 35 | 0.81645 | 1.163759 | 1 |
| HALLMARK_XENOBIOTIC_METABOLISM | 0.31441048 | 198 | 0.699175 | 1.150249 | 1 |
| HALLMARK_UV_RESPONSE_UP | 0.125 | 155 | 0.599276 | 1.136887 | 0.9761724 |
| HALLMARK_REACTIVE_OXIGEN_SPECIES_PATHWAY | 0.22 | 46 | 0.848599 | 1.11999 | 0.97129846 |
| HALLMARK_HEME_METABOLISM | 0.2646503 | 191 | 0.597934 | 1.101115 | 0.72334665 |
| HALLMARK_ADIPOGENESIS | 0.24584104 | 195 | 0.628668 | 1.098276 | 0.6762306 |
| HALLMARK_MITOTIC_SPINDLE | 0.26055047 | 198 | 0.562246 | 1.077838 | 0.73321736 |
| HALLMARK_FATTY_ACID_METABOLISM | 0.26741996 | 157 | 0.586373 | 1.034118 | 0.8529015 |
| HALLMARK_APICAL_JUNCTION | 0.32026145 | 195 | 0.533531 | 1.023842 | 0.8157521 |
| HALLMARK_APICAL_SURFACE | 0.30769232 | 44 | 0.633561 | 1.01972 | 0.76893455 |
| HALLMARK_DNA_REPAIR | 0.4385965 | 143 | 0.565447 | 1.005199 | 0.7779733 |
| HALLMARK_MYOGENESIS | 0.57918555 | 199 | 0.538736 | 0.956211 | 0.91874576 |
| HALLMARK_COMPLEMENT | 0.6229261 | 195 | 0.500121 | 0.955069 | 0.88064915 |
| HALLMARK_OXIDATIVE_PHOSPHORYLATION | 0.4385965 | 198 | 0.650016 | 0.951478 | 0.84035134 |
| HALLMARK_E2F_TARGETS | 0.5671642 | 198 | 0.641151 | 0.945224 | 0.8144215 |
| HALLMARK_MTORC1_SIGNALING | 0.5260223 | 198 | 0.562164 | 0.926194 | 0.82797027 |
| HALLMARK_NOTCH_SIGNALING | 0.7170213 | 32 | 0.469431 | 0.856663 | 0.9837505 |
| HALLMARK_CHOLESTEROL_HOMEOSTASIS | 0.776699 | 73 | 0.444428 | 0.855078 | 0.9440868 |
| HALLMARK_G2M_CHECKPOINT | 0.7185501 | 198 | 0.583791 | 0.836091 | 0.9330952 |
| HALLMARK_BILE_ACID_METABOLISM | 0.73491377 | 112 | 0.473109 | 0.736843 | 1 |
| HALLMARK_MYC_TARGETS_V2 | 0.85532993 | 58 | 0.56871 | 0.693345 | 0.98105043 |

**Supplementary Table 2. RNA-seq analyses of the expression of 18 IRDS genes among three ARCaP sublines.**

| SYMBOL | ARCaP-Fast | | ARCaP-F11 | | ARCaP-B5 | |
|---|---|---|---|---|---|---|
| | #1 | #2 | #1 | #2 | #1 | #2 |
| HERC6 | 4.49 | 3.61 | 21.78 | 21.27 | 3.32 | 1.56 |
| IFI35 | 31.38 | 17.03 | 141.94 | 130.35 | 17.60 | 18.57 |
| IFI44 | 9.43 | 8.77 | 127.44 | 110.95 | 5.35 | 2.30 |
| IFI44L | 0.10 | 0.09 | 31.56 | 24.25 | 0.00 | 0.00 |
| IFI6 | 43.78 | 38.08 | 758.43 | 409.52 | 8.77 | 14.59 |
| IFIT1 | 13.46 | 13.98 | 141.94 | 121.02 | 18.46 | 8.17 |
| IFIT3 | 36.17 | 36.70 | 121.07 | 121.73 | 20.32 | 17.60 |
| IFITM1 | 232.70 | 173.10 | 478.44 | 414.74 | 49.86 | 295.20 |
| LAMP3 | 3.06 | 3.92 | 11.60 | 9.94 | 3.87 | 3.09 |
| LGALS3BP | 132.93 | 119.13 | 367.33 | 334.67 | 107.54 | 120.59 |
| MX1 | 14.90 | 9.35 | 310.43 | 242.32 | 2.38 | 1.61 |
| MX2 | 3.65 | 2.81 | 83.37 | 55.98 | 0.53 | 0.54 |
| OAS1 | 49.19 | 46.94 | 175.29 | 164.83 | 13.38 | 22.80 |
| OAS3 | 58.60 | 52.72 | 230.60 | 223.30 | 59.22 | 42.95 |
| OASL | 18.29 | 27.18 | 55.90 | 65.06 | 14.17 | 11.49 |
| PLSCR1 | 55.92 | 47.67 | 105.25 | 100.17 | 13.58 | 22.44 |
| STAT1 | 45.17 | 40.59 | 265.54 | 256.62 | 31.23 | 31.21 |
| USP18 | 1.46 | 1.40 | 31.96 | 24.73 | 1.37 | 1.14 |

**Supplementary Table 3. Tumor size of sub-cutaneous injected DU145-shCon and DU145-shIFIT5 lines.**

| Cell number | 1X10$^6$ | | 1X10$^4$ | | 1X10$^2$ | |
|---|---|---|---|---|---|---|
| | shCon | shIFIT5 | shCon | shIFIT5 | shCon | shIFIT5 |
| Tumor size (mm$^3$) | 466.80 | 65.40 | 143.60 | 49.40 | 190.40 | 0.00 |
| | 296.50 | 33.50 | 128.10 | 49.40 | 98.10 | 0.00 |
| | 222.30 | 26.20 | 125.50 | 33.50 | 71.90 | 0.00 |
| | 205.90 | 25.10 | 94.10 | 26.20 | 47.70 | 0.00 |
| | 147.10 | 25.10 | 65.40 | 25.10 | 0.00 | 0.00 |
| | 110.50 | 16.70 | 65.40 | 16.70 | 0.00 | 0.00 |
| | 87.00 | 14.10 | 65.40 | 14.10 | 0.00 | 0.00 |
| | 65.40 | 0.00 | 63.30 | 0.00 | 0.00 | 0.00 |
| Average (mm$^3$) | 200.18±132.41 | 25.76±18.95 | 93.85±33.83 | 26.80±17.12 | 51.01±68.14 | 0.00±0.00 |
| P value | 0.003620 | | 0.000240 | | 0.026303 | |

**Supplementary Table 4. Tumor weight of sub-cutaneous injected DU145-shCon and DU145-shIFIT5 lines.**

| Cell number | $1X10^6$ | | $1X10^4$ | | $1X10^2$ | |
|---|---|---|---|---|---|---|
| | shCon | shIFIT5 | shCon | shIFIT5 | shCon | shIFIT5 |
| Tumor weight (g) | 0.75 | 0.10 | 0.48 | 0.09 | 0.16 | 0.00 |
| | 0.68 | 0.09 | 0.43 | 0.08 | 0.08 | 0.00 |
| | 0.67 | 0.08 | 0.32 | 0.06 | 0.05 | 0.00 |
| | 0.61 | 0.07 | 0.25 | 0.03 | 0.03 | 0.00 |
| | 0.28 | 0.04 | 0.14 | 0.03 | 0.00 | 0.00 |
| | 0.26 | 0.03 | 0.13 | 0.03 | 0.00 | 0.00 |
| | 0.21 | 0.02 | 0.12 | 0.02 | 0.00 | 0.00 |
| | 0.14 | 0.00 | 0.06 | 0.00 | 0.00 | 0.00 |
| Average (g) | 0.45±0.25 | 0.06±0.03 | 0.24±0.16 | 0.05±0.03 | 0.08±0.06 | 0.00±0.00 |
| P value | 0.001522 | | 0.005140 | | 0.040319 | |

**Supplementary Table 5. The information of primer sequences**

| Gene Name | Primer direction | Primer sequence |
|---|---|---|
| 18S | Forward | GGAATTGACGGAAGGGCACCACC |
| | Reverse | GTGCAGCCCCGGACATCTAAGG |
| STAT1 | Forward | ATGGCAGTCTGGCGGCTGAATT |
| | Reverse | CCAAACCAGGCTGGCACAATTG |
| IFIT5 | Forward | TAAAAAAGGCCTTGGAGGTG |
| | Reverse | CCAGGTCTGTGTAGGCAAAT |
| SOX2 | Forward | AGCTACAGCATGATGCAGGA |
| | Reverse | GGTCATGGAGTTGTACTGCA |
| BMI1 | Forward | AATCCCCACCTGATGTGTGT |
| | Reverse | GCTGGTCTCCAGGTAACGAA |
| NANOG | Forward | CATGAGTGTGGATCCAGCTTG |
| | Reverse | CCTGAATAAGCAGATCCATGG |
| IFITM1 | Forward | GGCTTCATAGCATTCGCCTACTC |
| | Reverse | AGATGTTCAGGCACTTGGCGGT |
| IFI6 | Forward | TGATGAGCTGGTCTGCGATCCT |
| | Reverse | GTAGCCCATCAGGGCACCAATA |
| MX1 | Forward | GGCTGTTTACCAGACTCCGACA |
| | Reverse | CACAAAGCCTGGCAGCTCTCTA |
| MX2 | Forward | AAAAGCAGCCCTGTGAGGCATG |
| | Reverse | GTGATCTCCAGGCTGATGAGCT |
| OAS3 | Forward | CCTGATTCTGCTGGTGAAGCAC |
| | Reverse | TCCCAGGCAAAGATGGTGAGGA |
| OAS1 | Forward | AGGAAAGGTGCTTCCGAGGTAG |
| | Reverse | GGACTGAGGAAGACAACCAGGT |
| IFI44 | Forward | GTGAGGTCTGTTTTCCAAGGGC |
| | Reverse | CGGCAGGTATTTGCCATCTTTCC |
| IFI44L | Forward | TGCACTGAGGCAGATGCTGCG |
| | Reverse | TCATTGCGGCACACCAGTACAG |
| IFIT1 | Forward | GCCTTGCTGAAGTGTGGAGGAA |
| | Reverse | ATCCAGGCGATAGGCAGAGATC |
| IFIT3 | Forward | CCTGGAATGCTTACGGCAAGCT |
| | Reverse | GAGCATCTGAGAGTCTGCCCAA |
